# Dopamine receptors in a songbird brain

**DOI:** 10.1002/cne.22255

**Published:** 2010-03-15

**Authors:** Lubica Kubikova, Kazuhiro Wada, Erich D Jarvis

**Affiliations:** 1 Department of Neurobiology, Howard Hughes Medical Institute, Duke University Medical Center,Durham, North Carolina 27710; 2 Department of Physiology and Ethology, Institute of Animal Biochemistry and Genetics, Slovak Academy of Sciences90028 Ivanka pri Dunaji, Slovakia; 3 Division of Integrated Life Science, Hokkaido University060-0810 Sapporo, Japan

**Keywords:** catecholamine, song nuclei, egr1, ZENK, D1, D2, zebra finch, chicken

## Abstract

Dopamine is a key neuromodulatory transmitter in the brain. It acts through dopamine receptors to affect changes in neural activity, gene expression, and behavior. In songbirds, dopamine is released into the striatal song nucleus Area X, and the levels depend on social contexts of undirected and directed singing. This differential release is associated with differential expression of activity-dependent genes, such as egr1 (avian zenk), which in mammalian brain are modulated by dopamine receptors. Here we cloned from zebra finch brain cDNAs of all avian dopamine receptors: the D1 (D1A, D1B, D1D) and D2 (D2, D3, D4) families. Comparative sequence analyses of predicted proteins revealed expected phylogenetic relationships, in which the D1 family exists as single exon and the D2 family exists as spliced exon genes. In both zebra finch and chicken, the D1A, D1B, and D2 receptors were highly expressed in the striatum, the D1D and D3 throughout the pallium and within the mesopallium, respectively, and the D4 mainly in the cerebellum. Furthermore, within the zebra finch, all receptors, except for D4, showed differential expression in song nuclei relative to the surrounding regions and developmentally regulated expression that decreased for most receptors during the sensory acquisition and sensorimotor phases of song learning. Within Area X, half of the cells expressed both D1A and D2 receptors, and a higher proportion of the D1A-only-containing neurons expressed egr1 during undirected but not during directed singing. Our findings are consistent with hypotheses that dopamine receptors may be involved in song development and social context-dependent behaviors. J. Comp. Neurol. 518:741–769, 2010. © 2009 Wiley-Liss, Inc.

The neurotransmitter dopamine plays an important role in motor control, modulation of motivated behaviors, and formation of memories (for review see Wise, 2004). Midbrain dopaminergic neurons (see Fig. 1, orange) send profuse projections to the striatum and sparser projections to the pallium (see Fig. 1, purple and green), including to the mammalian cortex (Kitt and Brauth, 1986; Durstewitz et al., 1999). When dopamine is released from presynaptic terminals of dopaminergic neurons, the dopamine binds to diverse sets of postsynaptic dopamine receptors that are classified into two families, D1 and D2 (Kebabian and Calne, 1979). Generally, D1 receptors activate and D2 receptors inhibit neural activity, adenylate cyclase, and downstream signaling pathways (Stoof and Kebabian, 1984; Gerfen, 2000). The D1 family includes D1A (also called D1), D1B (also called D5), D1C, and D1D receptors; the D2 family includes D2, D3, and D4 receptors (Bunzow et al., 1988; Dearry et al., 1990; Sokoloff et al., 1990; Sunahara et al., 1990,^1991^; Zhou et al., 1990; Grandy et al., 1991; Martens et al., 1991; Van Tol et al., 1991; Macrae and Brenner, 1995; Cardinaud et al., 1997; Hirano et al., 1998). D1C is not found in eutherian mammals, and D1D is thought to be avian specific (Demchyshyn et al.,1995).

**Figure 1:**
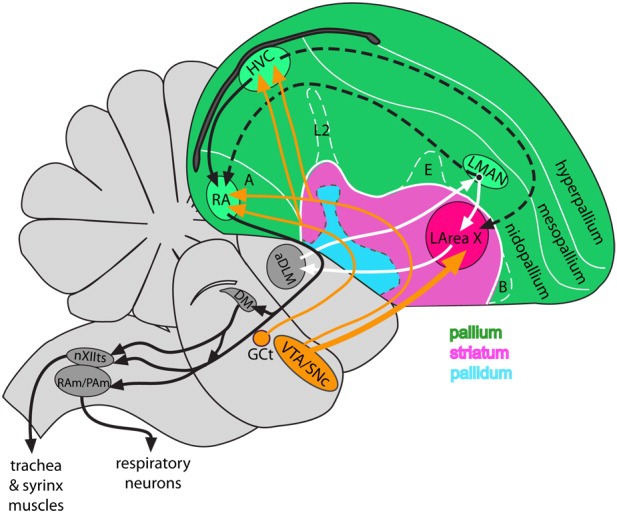
Diagram of the avian brain highlighting pallial, striatal, and pallidal telencephalic areas and the song system of songbirds. Black arrows, posterior vocal pathway; white arrows, anterior vocal pathway; dashed arrows, connections between the two pathways; orange arrows, dopaminergic (GCt and VTA-SNc) input into the song nuclei. For abbreviations see list.

In birds, autoradiographic ligand binding and kinetic studies revealed that proteins of both receptor families are expressed in the brain (Richfield et al., 1987; Dietl and Palacios, 1988; Casto and Ball, 1994; Ball et al., 1995; Demchyshyn et al., 1995; Schnabel and Braun, 1996; Stewart et al., 1996; Schnabel et al., 1997; Kostal et al., 1999; Kubikova et al., 2009). However, it is not possible from these studies to determine expression of the specific receptor types. Specific expression of several receptors as determined by in situ hybridizations revealed that the mRNAs of the D1A and D1B receptors are abundant in the chicken striatum, and D1B is also enriched in the mesopallium (Sun and Reiner, 2000; terminology from Reiner et al., 2004a). The mRNA expression for the D2 receptor has been characterized in the turkey brain (Schnell et al., 1999), but its distribution did not correspond with the D2 ligand protein binding distribution known in birds (Richfield et al.,1987; Dietl and Palacios, 1988; Schnabel and Braun, 1996; Stewart et al., 1996; Kostal et al., 1999). The mRNA distribution was restricted to several pallial areas (mesopallium and caudal nidopallium), but the ligand binding distribution was mainly in the striatum, as it is in the mammalian brain (Mansour et al.,1990; Weiner et al., 1991). Expression of the D1D, D3, and D4 receptors has not been characterized in the avian brain.

For songbirds, multiple researchers have hypothesized that the dopaminergic system is involved in the modulation of social-context-dependent song production and song learning (Jarvis et al., 1998; Hessler and Doupe, 1999; Ding and Perkel, 2002,^2004^; Gale and Perkel, 2005; Sasaki et al., 2006; Schroeder and Riters, 2006; Yanagihara and Hessler, 2006; Hara et al., 2007; Heimovics and Riters, 2008; Heimovics et al., 2009). Song production and learning are controlled by a network of interconnected vocal nuclei, also called *song nuclei*, distributed in two main pathways: 1) an anterior vocal pathway that forms a loop between LMAN in the pallium, Area X in the striatum, and DLM in the thalamus and that is necessary for song learning and juvenile subsong production and 2) a posterior vocal pathway that forms a projection from HVC to RA to brainstem motor neurons and that is necessary for song production (see Fig. 1; Nottebohm et al., 1976; Bottjer, 1987; Sohrabji et al., 1990; Scharff and Nottebohm, 1991; Nordeen and Nordeen, 1993; Aronov et al., 2008). When songbirds sing, the song nuclei show increased expression of immediate early genes (IEGs), such as egr1 (Jarvis and Nottebohm, 1997; Wada et al., 2006); egr1 expression in the mammalian brain is modulated by dopamine receptors (Gerfen, 2000). The singing-driven egr1 expression occurs throughout the song system nuclei when zebra finches produce undirected song but is low in the lateral part of the anterior pathway song nuclei and in the motor output nucleus RA when they produce directed song to another bird (Jarvis et al., 1998); undirected song is thought to be used for practice, whereas directed song is usually produced to a female during courtship (Sossinka et al., 1975; Jarvis et al., 1998; Kao et al., 2005; Ölveczky et al., 2005; Kao and Brainard, 2006). An opposite result of differential down-regulation in different social context occurs for the FoxP2 gene (Teramitsu and White, 2006).

We have proposed that the social context modulation of egr1 expression is controlled by dopaminergic input from the ventral tegmental area (VTA) and substanstia nigra pars compacta (SNc) onto presumed dopamine receptors in Area X (Jarvis et al., 1998). In this regard, the VTA-SNc has higher rates of increased neural activity, egr1 gene expression, and dopamine release into lateral Area X (LArea X) during directed relative to undirected singing (Sasaki et al., 2006; Yanagihara and Hessler, 2006; Hara et al., 2007). Furthermore, although the VTA-SNc is not required for the social context differences in LArea X, it is required for high levels of singing-induced egr1 induction in LArea X and for the social context differences in LMAN and RA (Hara et al., 2007). These findings are consistent with the known presence of high levels of dopamine, its synthetic enzyme tyrosine hydroxylase, and D1-like ligand receptor binding in Area X (Lewis, 1981; Barclay and Harding, 1988; Sakaguchi and Saito, 1989; Barclay et al., 1992; Bottjer, 1993; Casto and Ball, 1994; Soha et al., 1996; Harding et al., 1998; Appeltants et al., 2000). They are also consistent with pharmacological studies showing that D1-like and D2-like receptors in zebra finch Area X display excitatory and inhibitory physiological responses similar to those of D1-like and D2-like receptors in mammalian brain (Ding and Perkel, 2002,^2004^; Gale and Perkel, 2005). However, nothing is known about the specific dopamine receptors or their possible developmental regulation in the song nuclei.

To characterize dopamine receptors in the avian brain and address hypotheses on social context-dependent gene regulation in songbirds, we cloned zebra finch cDNAs of the D1A, D1B, avian-specific D1D, D2, D3, and D4 receptors and characterized their protein coding sequences and brain distribution profiles in zebra finches and chickens, their expression in song nuclei, their expression during song development, and the receptor neuron types that show differential gene regulation during singing in different social contexts in zebra finches.

## MATERIALS AND METHODS

### Animals

We used 33 zebra finches (*Taeniopygia guttata*) and three chickens for this study. The zebra finches were adult males (more than 120 days old; n = 13 total), adult females (n = 3), and juvenile males (n = 17). The chickens were adult males. The breakdown of experimental groups is described in the Behavior section. All animal protocols were approved by the Duke University Institutional Animal Care and Use Committee.

### Cloning of dopamine receptors and identification of full-length coding sequences

We attempted to clone cDNAs of dopamine receptors from zebra finch brain mRNA using degenerate primers for D1 and D2 family receptors. We chose primers that would amplify regions with sequences specific to a given receptor, to prevent cross-hybridization among receptors in the in situ hybridizations. For the D1 family (D1A, D1B, and D1D), we chose conserved regions from human, rat, chicken, and/or *Xenopus* sequences in Genbank (Sugamori et al., 1994; Demchyshyn et al., 1995; Sun and Reiner, 2000). For the D2 family (D2, D3, and D4), we chose conserved regions from human, mouse, rat, bovine, and/or *Xenopus* sequences in Genbank. For D4, we tried four different degenerate primer combinations, but they did not work (not shown). Instead, at the completion of our study, we used sequences of a partially sequenced D4 receptor in the recently released zebra finch genome (UCSC browser and NCBI accession No. XM_002196640; Warren et al, in preparation) and of a full-length great tit songbird (*Parus major*) cDNA sequence (accession No. DQ006802; Fidler et al., 2007) to generate nondegenerate primers against the putative full-length coding DNA sequence of the zebra cDNA. The sequences of the degenerate (for D1A, D1B, D1D, D2, and D3) and nongenerate (for D4) forward and reverse primers are given in Table 1. RT-PCR was performed on total RNA from adult zebra finch brains with the appropriate primer pairs. PCR products were examined on 1.5% agarose gels, and if predicted sized bands were present, they were cut from the gel and cloned into the pGEMTeasy plasmid (Promega, Madison, WI). These plasmids were transformed into XL-1 blue *Escherichia coli* supercompetent cells (Stragene, La Jolla, CA), and colonies with ampicillin (100 μg/ml) resistance were selected on agarose plates. From the colonies, DNA minipreps were performed. Inserts were 5′-and 3′-end sequenced and DNA sequences analyzed with BLASTN (DNA) and BLASTX (protein) GenBank searches to confirm cloning. All sequences have been deposited into GenBank (accession Nos. in Table 1).

**Table 1:** Accession Numbers and Primers^1^

Gene Tr	Accession No.	Forward primer	Reverse primer	Length (bp)
D1A	AB372107	5′-CCATCATGATAGTAACCTACA-3′	5′-GAATTAGCCCATCCAAACCA-3′	320
D1B	AB372108	5′-GTRGCCTTYGAYATYATGTGCTC-3′	5′-ARAANGGBARCCARCARCACAC-3′	625
D1D	AB372109	5′-GTRGCCTTYGAYATYATGTGCTC-3′	5′-ARAANGGBARCCARCARCACAC-3′	478
D2	AB490795 (for v1)	5′-TGYGCCATCAGCRTNGACAGGT-3′	5′-GCRCTRTTSACRTARCCHAGCCA-3′	851
D2	AB372110 (for v5)	5′-TGYGCCATCAGCRTNGACAGGT-3′	5′-GCRCTRTTSACRTARCCHAGCCA-3′	701
D3	AB372111	5′-TGYGCCATCAGCRTNGACAGGT-3′	5′-GCRCTRTTSACRTARCCHAGCCA-3′	780
D4	GQ359780	5′-ATCCTCCTCATCCTCCTTATCGT-3′	5′-ACGGTGTAAATGATGGGGTTGA-3′	687

1 Listed are the cloned dopamine receptor cDNA gene transcripts (Gene Tr), their accession Nos., the forward and reverse primers used to clone them, and the clone lengths. The primers for D1B and D1D as well for D2 v1, D2 v5, and D3 receptors were identical.

We used our partial dopamine receptor cDNA sequences to identify and predict the full-length coding sequences from the recently assembled sequenced zebra finch genome (assembled by the Genome Sequencing Center at the University of Washington St Louis and The UCSC Genome Browser http://genome.ucsc.edu/cgi-bin/hgGateway?db=taeGut1). The zebra finch cDNA fragments were BLAST searched against the zebra finch genome. The genomic region containing the hit sequence with the highest score was then scanned with GeneScan (Burge and Karlin, 1998; http://genes.mit.edu/GENSCAN.html) to generate predicted amino acid coding regions. We then performed quality control analyses, i.e., support by ESTs and homologies to other species, to generate curated protein coding sequences. While we were preparing this paper for submission, the ENSEMBLE group generated machine-predicted and annotated protein sequences from the zebra finch genome (ENSEMBLE set 54; Warren et al, in preparation), and we compared their dopamine receptor predictions (accession Nos. XP_002196692.1, XP_002193417.1, XP_002189224.1, XP_002191647.1, XP_002189176.1, XP_002196676.1 for D1A, D1B, D1D aka D1C, D2 variant 1, D3, and D4, respectively) with our cDNAs and curated predicted proteins (accession Nos. BR000842, BR000843, BR000844, BR000845 for D2 variant 1, BR000846, and BR000846, respectively). We created alignments across and within species using the European Bioinformatics Institute version of ClustalW (Higgins and Sharp,1988; http://www.ebi.ac.uk/Tools/clustalw2/index.html), Dialign (Morgenstern, 2004), or Genetyx (Zenetikkusu, Inc) software. We also used the nexus-formatted text output of these programs to generate phylogenetic trees with the online interactive tree of life (iTOL) software (http://itol.embl.de/index.shtml). To generate alignment summary diagrams, we used Microsoft Powerpoint and Word. To verify and identify transmembrane domains, we used the TMHMM program at the Center for Biological Sequences (http://www.cbs.dtu.dk/services/TMHMM/).

### Behavior

Zebra finch males were housed overnight in sound-isolation boxes. On the next morning, they were observed by audio and videotaped for at least 1 hour. Males that did not sing were taken as silent controls for the brain distribution study in adults (n = 3) and for developmental expression analyses in juveniles (n = 3 each age group of 15, 35, 45, and 75 days old, n = 5 of 25 days old). Adult females (n = 3) were treated in the same manner. For chickens, animals were taken while in their home cage. The animals were then killed, and brains were quickly dissected, frozen in OCT Compound (Sakura) in block molds, stored at −80°C, and used for single-label in situ hybridizations.

For the social context singing behavior study, adult male zebra finches were treated in a similar manner, except that males that sang at least 20 song bouts of undirected (n = 6) or directed (n = 4) song in 1 hour were taken. This amount of singing is known to induce high levels of egr1 protein expression in Area X and other song nuclei (Jarvis et al., 1998; Kubikova et al., 2007). A song bout is defined as a bout of singing, separated by at least 2 seconds of silence from another bout of song. For the undirected song group, birds were kept in the sound isolation chamber alone. For the directed song group, a female was placed in the cage before the lights were turned on. The female was separated from the male by a cage-wall barrier, which encourages more directed singing because of the lack of tactile contact with the female. After the 1-hour sessions, the males were perfused with 4% paraformaldehyde, and their brains were dissected, postfixed in the same solution, immersed in 20% and then in 30% sucrose, frozen in OCT compound in block molds, stored at −80°C, and used for double-and triple-labeling experiments. The brains were perfused first rather than fresh frozen, because fixation before freezing generates better morphology for cell identifications in double-and triple-labeling experiments.

### Single-, double-, and triple-label in situ hybridization and immunocytochemistry

Single-label radioactive in situ hybridizations (RISH) were done as previously described (Wada et al., 2004,^2006^). Fresh frozen sections (12 μm) were cut on a cryostat throughout the entire brain: left hemisphere in the sagittal plane and the right hemisphere in the coronal plane. Sections were mounted onto silanated glass slides and stored at −80°C. Corresponding sections of all birds were fixed in 4% paraformaldehyde in PBS (pH 7.4) and hybridized at 65°C with antisense ^35^S-UTP labeled riboprobes made from the zebra finch dopamine receptor cDNAs of this study. T7 and SP6 RNA polymerases were used to make the antisense and sense riboprobes, respectively. The hybridized sections were first exposed to X-ray film (Biomax MR; Kodak) for 1–4 days, then dipped into autoradiographic emulsion (NTB2; Kodak), incubated for 2–4 weeks at 4°C, processed with Kodak developer (D-19) and fixer, and Nissl stained with cresyl violet acetate solution (Sigma, St. Louis, MO). Films and slides with the same receptors were developed at the same time.

To perform double labeling in situ hybridization experiments with D1A and D2 receptors, a ^35^S-UTP labeled riboprobe was used for RISH for one dopamine receptor mRNA and a DIG-labeled riboprobe was used for fluorescent in situ hybridization (FISH) for the other dopamine receptor mRNA. Fixed frozen thick sections (30 μm) were cut in the sagittal plane on a cryostat and free-floated in PBS; we found that free-floating sections gave stronger signals for double-label detection than slide-mounted sections. To test for possible bias of the label, riboprobe types (^35^S and DIG) were switched for the receptor types (D2 and D1A, respectively) in a second experiment. The DIG-labeled riboprobes were prepared from 0.15 μg of the selected cDNA, 4 μl of 5× RNA transcription buffer, 2 μl of 10× DIG labeling mix (Roche), 1 μl of RNase inhibitor, 1 μl of RNA polymerase, and DEPC water (20 μl total volume). The reaction mixture was incubated for 2 hours at 37°C. Then, 30 μl of DEPC water was added, and the riboprobe was precipitated. Both riboprobes were simultaneously added to the same hybridization solution as used for single-label in situ hybridizations (Wada et al., 2004) to obtain final concentrations of 10^7^ cpm ^35^S probe and 1.5–3 μg of DIG probe per 1 ml hybridization solution. The riboprobes in solution were denatured for 6 minutes at 90°C. The free-floating sections were washed for 2 × 10 minutes in PBS, then 2 × 15 minutes in 0.75% glycine in PBS, and 20 minutes in 0.3% Triton X-100 in PBS and rinsed for 2 × 5 minutes in PBS, acetylated for 10 minutes in 0.25% acetic anhydride and 0.03% trietanolamine solution, rinsed for 3 × 5 minutes in PBS, and prehybridized for 45 minutes at 60°C and then 15 minutes at 70°C in the hybridization solution with the two riboprobes. Hybridization was done at 60°C for 16–18 hours; this is 5°C lower than for the single-label in situ, which helped to preserve tissue morphology and proteins for the immunocytochemistry. On the next day, the sections were washed for 2 × 5 minutes in 2× SSPE buffer, 30 minutes at 60°C in 2× SSPE buffer containing 50% formamide, 2 × 15 minutes at 60°C in 0.1× SSPE buffer and then cooled for 5 minutes at RT in 0.1× SSPE buffer and rinsed in PBS. Then, endogenous peroxidase activity was quenched in 1% H_2_O_2_ for 20 minutes, the sections were washed for 2 × 5 minutes in PBS and 1 × 5 minutes in TBS buffer (0.09% NaCl, 0.11% Tris, pH 7.4), and nonspecific binding was blocked for 30 minutes in TNB blocking buffer [1% blocking agent from TSA kit (Invitrogen, Carlsbad, CA) in TBS]. The sections were then incubated with the primary antibody anti-DIG-HRP (Roche, Indianapolis, IN; 1:100 in TNB) at 4°C overnight.

To perform triple-labeling experiments for D1A, D2, and egr1, the same procedure was applied, but a polyclonal rabbit anti-egr1 antibody (Santa Cruz Biotechnology, Santa Cruz, CA; catalog No. sc-189, 1:200) was added at the same time as the anti-DIG-HRP antibody. The antibody was raised against a 19-amino-acid peptide of the carboxy-terminus of the human egr-1 (sequence STGLSDMTATFSPRTIEIC). The specificity of a similar antibody raised against mouse egr1 that reciprocally cross-reacts with human egr1 protein (Santa Cruz Biotechnology) was determined in zebra finches and canaries (Mello and Ribeiro 1998), in which a single activity-induced protein in Western blots is seen in brain extracts. Preabsorbtion with the peptide to which the mouse antibody was raised completely eliminates the nuclear staining in tissue sections (Mello and Ribeiro, 1998). We BLAST searched the human peptide against Genbank and found that it recognizes only egr1 sequences at high identity (>80%), including a stretch of 16 amino acids of the zebra finch peptide sequence at 87% identity (LSDINSAFSPRTIEIC), derived from the zebra finch genome and cDNA egr1 sequences. This human egr1 antibody has been used in many published investigations of egr-1 immunoreactivity in the avian brain. After 3 × 3 minutes washes in TBS-T buffer, D1A or D2 mRNAs were visualized using a TSA kit with the fluorophore Alexa 488 or Alexa 594 (Invitrogen). The sections were washed for 3 × 3 minutes in TBS-T buffer, and the egr1 protein (i.e., antibody) was detected by 2 hours of incubation with a goat anti-rabbit IgG conjugated to Alexa 488 or Alexa 594 (Invitrogen; 1:200). The Alexa colors were alternated for mRNA and protein. The sections were washed for 3 × 3 minutes in TBS, mounted on slides, rinsed in deionized H_2_O, and dried. Then they were dipped in autoradiographic emulsion (NTB-2; Kodak), exposed for 3 days, and developed. They were coversliped with Vectashield solution containing DAPI (Vector Laboratories, Burlingame, CA).

### Quantification and statistics

For single-label in situ hybridizations, we quantified dopamine receptor expression levels from the X-ray films. X-ray film brain gene expression images were digitally scanned from a dissecting microscope connected to a Spot III CCD camera with Spot Advanced imaging software (Diagnostic Instruments, Sterling Heights, MI). Scion Image (NIH) was used to measure the mean pixel intensities in the brain areas of interest. The whole song nucleus or surrounding area was selected using the Freehand Selection tool in Scion Image. For HVC and RA, we chose HVC_shelf_ and RA_cup_ as the surrounding areas, respectively, for two reasons: 1) they are of interest as auditory regions (Jarvis and Nottebohm, 1997; Mello et al., 1998), and 2) their dopamine receptor gene expression was representative of the brain regions surrounding the respective song nuclei HVC and RA. HVC_shelf_ is the area ventrally adjacent to HVC, and RA_cup_ is on the ventral anterior side of RA. The borders of these regions are not well defined, so we measured the pixel density in smaller areas within these regions. The mean pixel density was calculated using the Analyse-Measure tool of Scion Image. The pixel density of the film immediately adjacent to the measured areas in the brain was quantified and subtracted from the pixel density in each song nucleus and the area surrounding each song nucleus. Three counts per brain region were taken consistently from three adjacent sections per animal.

For double-and triple-labeling experiments, images from Area X were acquired at ×40 magnification on a fluorescent microscope (Leica). D1A and D2 mRNA double labeling (^35^S and DIG) was assessed in a 100-× 100-μm field in the center of the image (n = 1,226 DAPI^+^ cells from n = 7 animals; average number of counted cells/bird = 190.4 ± 25.3). D1A and D2 mRNA with egr1 protein triple labeling was assessed in a 290-× 220-μm field (n = 747 total cells or average of 124.5 ± 29.5 egr1^+^ cells/undirected singing bird and 198 total cells or average of 49.5 ± 13.2 egr1^+^ cells/directed singing bird). We included a larger field of view for the triple labeling to increase cell sample size, because of the lower density of egr1 labeled cells relative to dopamine receptor labeled cells. The cells were considered labeled if the fluorescent signal surrounded (for dopamine receptors) or overlapped (for egr1) DAPI-labeled nuclei. In the case of RISH, the cells were considered labeled if there were at least five silver grains in the emulsion above the cell within a distance of two times the DAPI-defined radius of the nucleus (i.e., above DAPI-stained nucleus plus one radius). Furthermore, to confirm that the numbers of D1A and/or D2 labeled cells using RISH in single-and double/triple-labeling experiments were the same, we counted the number of labeled cells in the single-labeling experiment by using the same method, but at 63× magnification. Nissl staining instead of DAPI was used to localize cells in the single-labeling experiment. Three sections per animal were used, and the values were averaged for each animal. For the single-labeled sections, we quantified the number of D1A^+^ and D2^+^ cells also in anterior striatum (ASt) and the number of D1B^+^ cells in LArea X and ASt.

For statistical analyses of the adult expression study (see Fig. 11), all receptor types and brain areas were compared by paired *t*-test. The null hypothesis was that the ratio of expression in a song nucleus to its surrounding area is 1, that is, no different. To avoid finding significant differences found by chance in multiple paired *t*-tests, we used Bonferroni correction. The receptor types and song nuclei were selected as independent variables. In the developmental study (see Fig. 13), we compared the expression of all receptors for each brain area or each song nucleus as a ratio with its surrounding region in separate ANOVAs, because of the many tests needed for multiple time points. To avoid significant differences found by chance in multiple ANOVAs, we used Bonferroni correction. The null hypothesis was that the expression for the individual receptor in each brain area does not change during development. The ANOVAs were followed by Fisher's protected least significant difference (Fisher's PLSD) post hoc tests. The independent variable was the receptor type. To assess a possible bias of radioactive vs. DIG probes for D1A and D2 receptors, we compared the numbers of labeled cells in reverse probe experiments for the same animals using a paired *t*-test.

### Figure preparation

The photomicrographs were adjusted in Adobe Photoshop CS3. The Levels function was used to spread gray values or colors within the visible spectrum. Color images were further color balanced so that the colors of all sections are similar.

## RESULTS

### Comparative analyses of zebra finch dopamine receptor sequences

**Figure 2:**
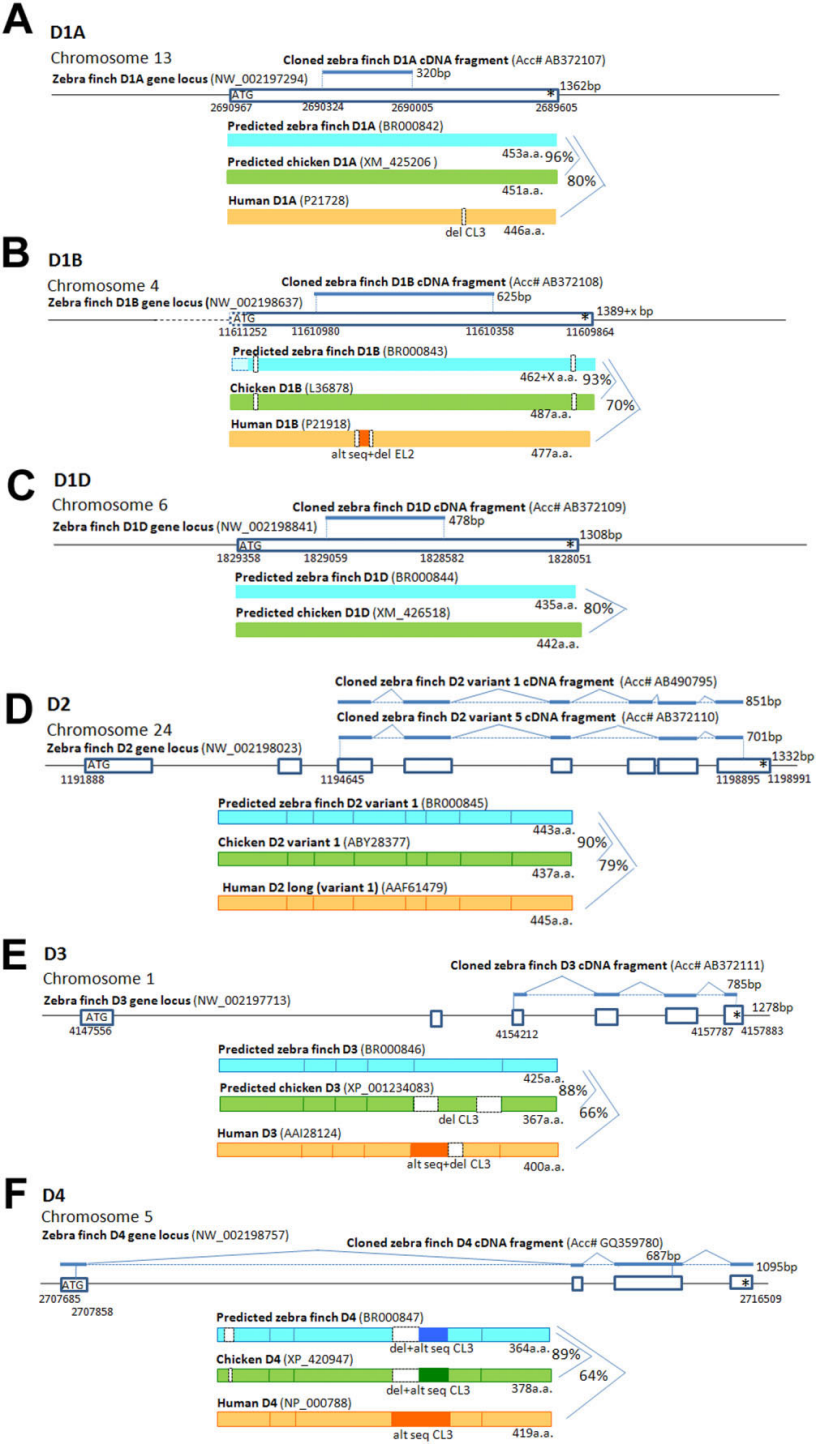
General structure and sequence comparisons of the six zebra finch dopamine receptors relative the homologous receptors in chicken and human. A–F: For each receptor, the size and position of the cloned zebra finch cDNA fragment (top solid blue lines) aligned to the predicted exon coding (blue open bars) and intron noncoding (black lines) sequence of the zebra finch genome are shown. Asterisk indicates a stop codon. The D1 genes have one coding exon each, D2 has eight, D3 has six, and D4 has four. Below the chromosomal sequences are comparisons of the genome-predicted or mRNA-derived protein coding sequences among zebra finch (turquoise), chicken (green), and human (orange bars). The percentage values indicate percentage identities between zebra finch and chicken or zebra finch and human. The lighter blue 5′ end of the zebra finch D1B protein indicates that this region has not been sequenced yet in the genome but is expected to be present because of the sequence found in the chicken and human genomes. The D4 zebra finch cDNA clone spans the full-length coding region, but the accession number refers to the 687 bp of cDNA sequence obtained to date; the remaining sequence is inferred from the zebra finch genome. White bars represent deletions (del); darker colored bars represent nonhomologous alternate sequences (alt seq). The predicted zebra finch proteins are those that we generated and curated with GENESCAN and the UCSC genome browser. We further curated the predicted ENSEMBLE chicken D3 protein sequence, becauise it had 300 more 5′ a.a. than all other D3 proteins in the database, which we believe was a computational error. Detailed protein alignments are shown in Supporting Information Figure 1. CL3, cytoplasmatic loop 3; EL2, extracellular loop 2.

We successfully cloned cDNAs of all six dopamine receptors from the zebra finch brain: D1A, D1B, D1D, D2, D3, and D4 (Fig. 2, top lines in each panel). By using these cDNA sequences, Genescan protein prediction software, and comparisons with other species, we identified the full-length coding regions of these dopamine receptors in the zebra finch genome (Fig. 2; detailed protein alignments in Supp. Info. Fig. 1). Consistently with findings in nonaves (Sunahara et al., 1990; Callier et al., 2003), we found that the zebra finch D1 family receptors exists as single exon genes, whereas the D2 family exists as spliced exons (Fig. 2). All receptors had the expected seven transmembrane domains (TM) of G-protein-coupled receptors (Supp. Info. Fig. 1).

Cross-species comparisons revealed relatively high protein sequence homologies of the zebra finch receptors with chicken (80–96%) and human (64–80%). The D1A receptor had the highest levels of homologies; relative to birds, humans had only a short 4-a.a. deletion in the third cytoplasmatic loop between TM domains 6 and 7 (Fig. 2A; Supp. Info. Fig. 1A). The D1B receptor had less homology mainly because of a different and shorter sequence in humans in the second extracellular loop between TM domains 4 and 5 (Fig. 2B; Supp. Info. Fig. 1B). The zebra finch D1D receptor had high homology (80%) only to the chicken D1D receptor. Relative to zebra finch, the chicken had a small deletion in the second extracellular loop and a longer carboxy terminal (Fig. 2C; Supp. Info. Fig. 1C). Some D1D sequence hits to chicken in the NCBI database were machine annotated as D1C (accession Nos. NP_001136143 and FJ432666.1). However, we found that these chicken protein sequences and our homologous zebra finch D1D did not have sufficiently higher identities to nonavian D1C sequences (frog and fish) than they had to D1A and D1B sequences of these same species [BLAST hits of the zebra finch D1D were 61%, 60%, and 57% to *Xenopus* D1C, D1B, and D1A, respectively; accession Nos. P42291, P42290, and P42289 (Sugamori et al., 1994)]. In addition, when we BLAST searched each zebra finch receptor against the zebra finch genome (UCSC browser) at a stringency that allowed cross-detection of D1-like or D2-like receptor genes within and sometimes across families, we were unable to find any other dopamine receptor than the six identified in this study. These findings suggest that the D1D and D1C annotations for chicken (and finch) are really the same gene. Further, the results support the conclusion that D1D is either unique to birds (Demchyshyn et al., 1995) or that D1D and D1C are homologs, but highly divergent (see discussion).

Relative to the D1 family, the cross-species comparisons revealed that the D2 family had lower homologies, which appeared to be due to splicing differences within and across species. We identified two splice variants of the D2 receptor in our cDNA products. The longest cDNA variant, which we call *variant 1*, matched with 100% identity the protein we predicted from the finch genome sequence (accession No. BR000845) and to variant 1 predicted by ENSEMBLE (XP_002191647; Supp. Info. Fig. 2A); it showed the closest cross-species homologies to chicken D2 variant 1 (90%) and human D2 variant “long” (79%; Fig. 2D; Supp. Info. Fig. 1D). Our other zebra finch variant, which we call *variant 5*, had a deletion within the third cytoplasmic loop between TM domains 5 and 6 (Supp. Info. Fig. 2A). Our D2 variant 5 was not found among the three additional zebra finch D2 variants 2, 3, and 4 predicted by ENSEMBLE, but all of these predicted variants had different deletions within the same third cytoplasmic loop (Supp. Info. Fig. 2A). We noted that this loop appeared to be alternatively spliced in cDNAs of chicken and humans (and other mammals), but these other species' variants also did not match our zebra finch variant 5. This suggests that alternative variants of the third cytoplasmic loop are relatively unique across distantly related species or that the homologous splice variants have not yet been identified across species. The zebra finch D3 receptor had lower levels of homologies to chicken and human D3 (88% and 66%), mainly because of variation also within the third cytoplasmic loop (Fig. 2E; Supp. Info. Fig. 1E). Relative to our zebra finch cDNA and our predicted D3 protein sequence (BR000846), the predicted chicken D3 had two deletions at the ends of the third cytoplasmic loop, whereas the human had an alternative but shorter sequence within the loop (Fig. 2E; Supp. Info. Fig. 1E). The D4 receptor also differed within the third cytoplasmic loop of all three species, where the zebra finch and chicken had similar lengths but alternative sequences, and humans had an alternative sequence that was nearly double the length of that in birds (Fig. 2F; Supp. Info. Fig. 1F). These findings indicate that, for the D2 receptor family, the third cytoplasmic loop between the fifth and sixth transmembrane domains can undergo large evolutionary changes.

**Figure 3:**
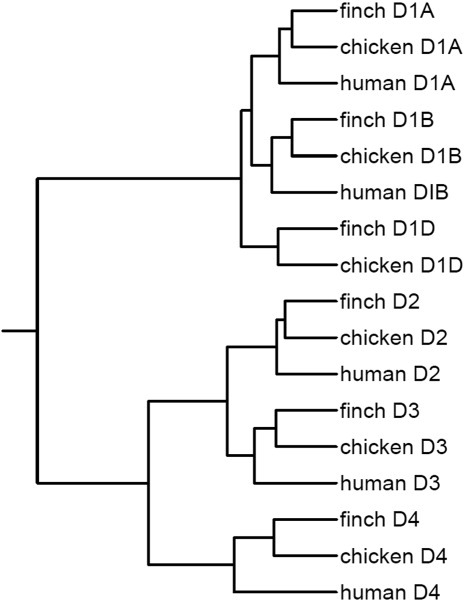
Phylogenetic analyses of dopamine receptors in the zebra finch, chicken, and human. Shown is a phylogram generated with the full-length protein coding sequences (Supp. Info. Fig. 1), the dialign alignments (http://bibiserv.techfak.uni-bielefeld.de/dialign/submission.html), and the iTOL tree-generating software (http://itol.embl.de/). For D2 and D3, variant 1 sequences were used. Branch lengths represent evolutionary time separating gene relationships (longer branch, more time). The D1 family has shorter branch lengths, indicating that they are probably more closely related than the D2 family. All receptor types show closer homologies to each other across species than they do to other receptor types within species.

Phylogram analysis confirmed species and gene relationships, where the D1 receptors (D1A, D1B, and D1D) clustered closer among each other across species and as a family and the D2 receptors (D2, D3, and D4) among each other across species and as a family (Fig. 3). This analysis further revealed 1) that the D1A and D1B receptors are more closely related to each other than they are to the avian specific D1D receptor; 2) that the D1 receptor family (D1A, D1B, and D1D) genes are more closely related to each other (shorter branch lengths) than the D2 family (D2, D3, and D4) are to each other (longer branch lengths; Fig. 3), in part as a result of the large differences in the third cytoplasmic loop in the D2 family; and 3) that, among the D2 family, D2 and D3 are more closely related to each other than they are to D4 (Fig. 3).

We performed similar analyses on the cloned partial cDNA sequences to address whether these receptors could cross-hybridize to each other in in situ hybridization experiments. We found that the cDNA fragments of each gene were less than 67% identical to the predicted full-length transcripts of any of the other receptors, except for the D1D cDNA fragment against the D1B transcript (74%; Table 2). However, none of them met our threshold of ∼85% identity necessary to cross-hybridize under our in situ hybridization conditions (Mello et al., 1997; Wada et al., 2004,^2006^; and unpublished observations). Thus, as intended in our PCR cloning design, the cloned cDNA fragments we have obtained should detect expression patterns specific for each dopamine receptor.

**Table 2:** Cross-Dopamine Receptor cDNA Sequence Identities^1^

	D1AFrag	D1BFrag	D1DFrag	D2v1Frag	D3Frag	D4cDNA
D1AFull	100	60	55	5	17	11
D1BFull	67	100	74	15	5	17
D1DFull	50	22	100	20	12	13
D2v1Full	5	12	13	100	61	31
D3Full	17	4	5	61	100	24
D4Full	12	5	9	31	24	100

1 Shown are the percentage sequence identities of each cloned dopamine cDNA fragment (D1A, D1B, D1D, D2, D3) and full-length cDNA (D4) to the full-length transcript of all the dopamine receptors. Because the full-length transcript is expected to be expressed in cells, the identities indicate cross-in situ hybridization potential of each receptor cDNA fragment to the full-length transcript of the other five receptors. All cross-cDNA identities are below our 85% cutoff.

### Dopamine receptor expression patterns in the avian brain

**Figure 4:**
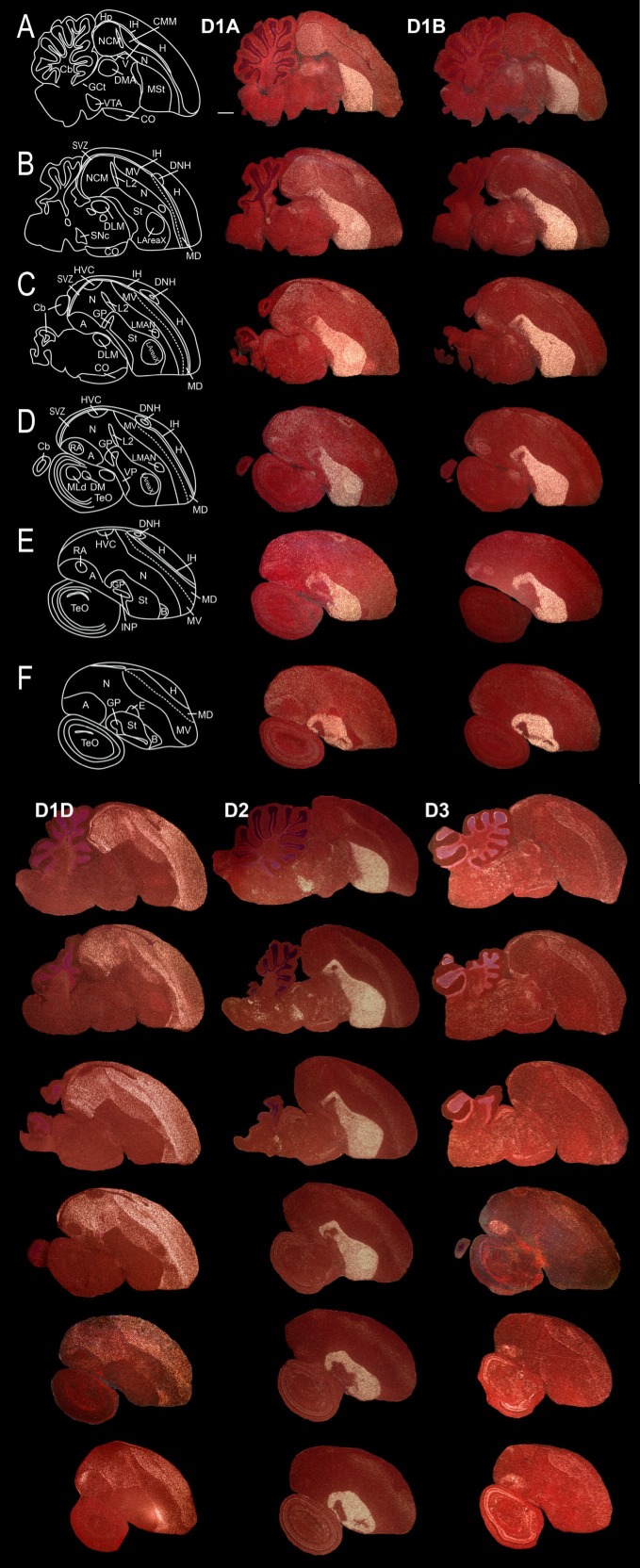
Expression profiles of dopamine receptor types in sagittal series from adult male zebra finch brain. A–F: Rows showing medial to lateral series across two pages of this report with the respective drawings on the left. Columns are labeled on the top for each receptor (D4 pattern is shown separately in Fig. 6). The images were taken under darkfield microscopy. White silver grains, dopamine receptor mRNA expression; red, cresyl violet stain. Rostral is right, dorsal is upward. The sequences of the cDNA probes used are in Genbank (accession Nos. AB372107, AB372108, AB372109, AB3490795, AB327111, for D1A, D1B, D1D, D2 transcript variant 1, and D3 respectively; Table 1). Scale bar = 1 mm.

**Figure 5:**
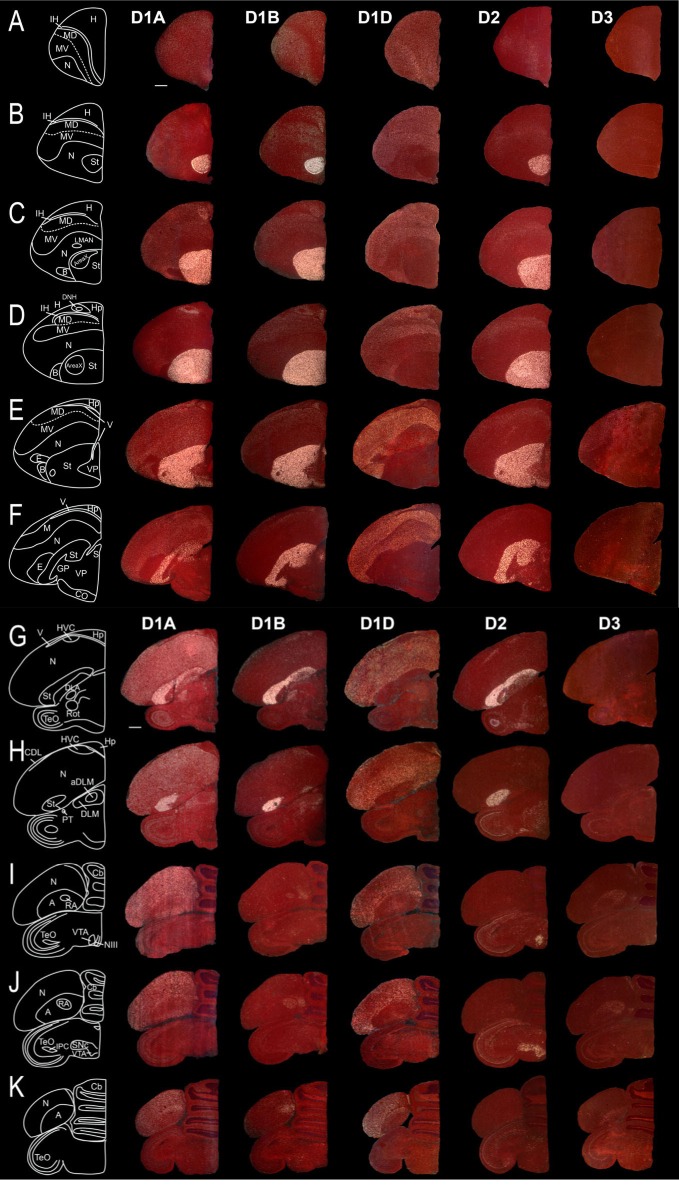
Expression profiles of dopamine receptor types in frontal series of sections of one brain hemisphere of an adult male zebra finch. A–K: Rows showing rostral to caudal series with the corresponding drawings on the left. Columns are labeled on the top for each receptor. The images were taken under darkfield microscopy. White silver grains, dopamine receptor mRNA expression; red, cresyl violet stain. Dorsal is upward, medial is right. Scale bar = 1 mm.

**Figure 6:**
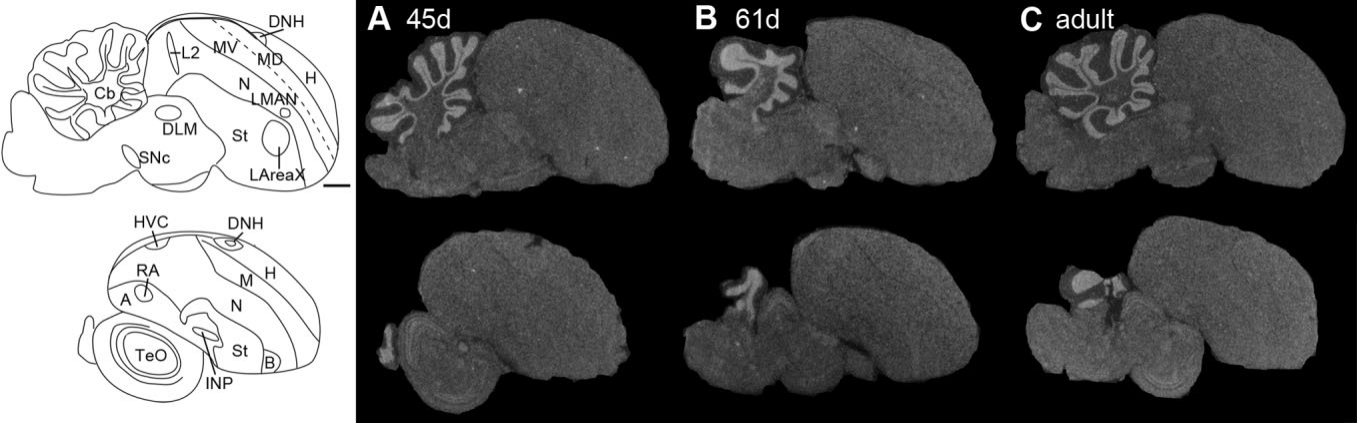
Expression profile of the D4 dopamine receptor in the sagittal plane from male zebra finch brains during two developmental ages in days (d; A,B) and in adulthood (C). Images were taken from film autoradiograms and inverted. Only several sections are shown, because there was not much differential expression of D4 receptor in the telencephalon. White, dopamine receptor mRNA expression. Dorsal is upward, rostral is right. Genbank accession number of probe sequence is GQ359780 (Table 1). Scale bar = 1 mm.

Within the zebra finch telencephalon, each receptor had a unique expression pattern: D1A, D1B, and D2 (variant 1) were highly enriched in the striatum (a subpallial structure); D1D and D3 were enriched in different subdivisions of the pallium (sagittals in Fig. 4; frontals in Fig. 5); and D4 was barely expressed in the brain, except for the cerebellum (Fig. 6).

**Figure 7:**
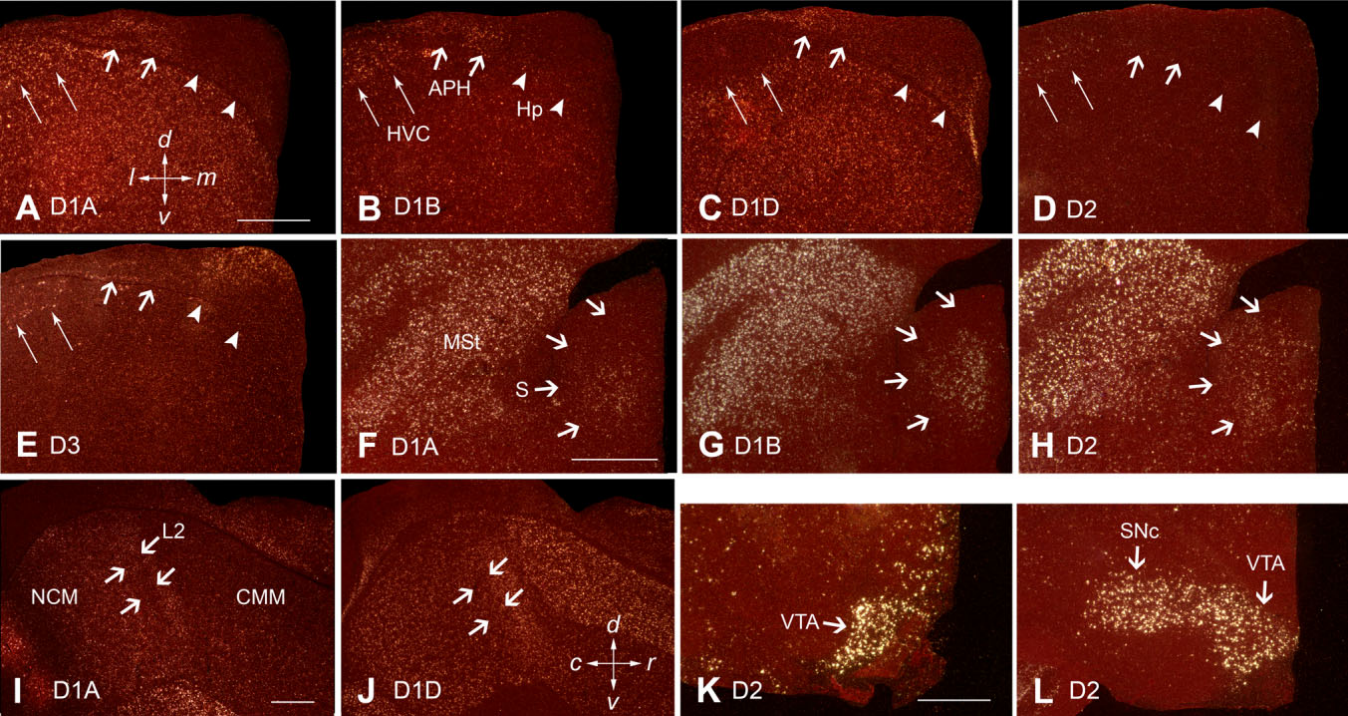
Higher power images of differential dopamine receptor subtype expression in specific zebra finch brain regions. A–E: Hippocampal formation. F–H: Septum. I,J: Field L2, NCM, and CMM. K,L: Midbrain dopaminergic cell groups VTA and SNc. Arrowheads in A–E point to the ventricle. All sections are coronal, except for I and J, which are sagittal. White silver grains, dopamine receptor mRNA expression; red, cresyl violet stain. Scale bars = 0.5 mm in A (applies to A–E); 0.5 mm in F (applies to F–H); 0.5 mm in I (applies to I,J); 0.5 mm in K (applies to K,L).

Within the subpallium, high D1A, D1B, and D2 mRNA levels were also present in the intrapeduncular nucleus (INP; Fig. 4E) and within the medial part of the septum (Figs. 5F, 7FF–7H). This is consistent with the hypothesis that the INP and medial septum are developmentally related to the striatum (Reiner et al., 2004a). Isolated cells with high levels of the D1A, D1B, D2, and D3 receptors were found in the pallidum (GP and VP; Figs. 4E4F, 5E,5F), the homologue of the mammalian globus pallidus and ventral pallidum.

Within the pallium, D1A receptors were lowest in sensory thalamic recipient zones: L2 for auditory input, entopallium (E) for visual input, and basorostralis (B) for somatosensory input (Figs. 4, 5E,5F, 7I). D1B was higher in the caudomedial part of the arcopallium (Figs. 4C, 5I–5K) and throughout the mesopallium (Figs. 4, 5A–5F). The D1A and D1B receptors were high locally around the DNH nucleus in the hyperpallium (Figs. 4B–5E, 5D); DNH shows activation during night vision in migratory songbirds (Mouritsen et al., 2005). D1D showed gradients of expression, including within the nidopallium culminating in high levels in B but low levels in E and L2 (Figs. 4, 5, 7J) and within the intermediate arcopallium around the song nucleus RA (Figs. 4D, 5J). All D1 receptor subtypes were higher in the parahipocampal area (APH; Fig. 7A–7C). The D2 receptor had a gradient of expression in the dorsal and ventral mesopallium, with the higher expression levels at the lamina between the two halves of the mesopallium. The D3 receptor expression was mainly restricted to the mesopallium (Figs. 4, 5), the intermediate arcopallium (Fig. 4E,4F), in a band of cells in the subventricular zone between the ventrical and hippocampus (Figs. 4A–4C, 7E), and within the dorsal hippocampus (Fig. 7E).

Within the brainstem, five (D1A, D1B, D2, D3, and D4) of the six receptors were expressed with specific patterns (Figs. 4–7); the exception was D1D, which appeared to have little if any expression in the brainstem. D1A and D1B were enriched within dorsal thalamic nuclei, whereas D3 was enriched in their shell regions (Figs. 4A, 5H), which receive feedback projections from the telencephalon (Mello et al., 1998). Four of the receptors (D1A, D2, D3, and D4) were expressed in midbrain regions, such as the optic tectum (Figs. 4D–4F, 5H–5K, 6). Notable differences were within the midbrain dopamine-synthesizing cell groups, the VTA-SNc and GCt. In these cell groups, D2 was highly expressed, D1B was low, and the other receptors appeared undetectable (Figs. 4A,B, 5IJ, 7K,L).

**Figure 8:**
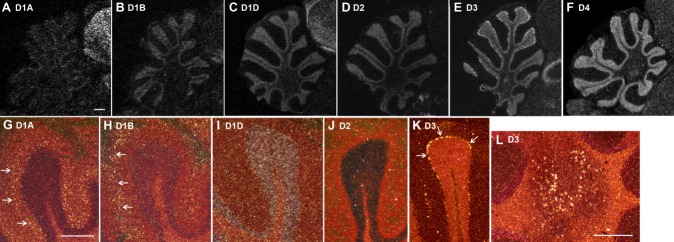
Higher power images of differential dopamine receptor subtype expression in the cerebellum. A–F show film autoradiogram images that were inverted, where expression can be seen in the granular layer for all receptors; the Nissl staining of the dense granular layer in the in situ hybridizations masks the label. G–L show Nissl-stained images in darkfield, in which the differential expression (white silver grains) of several receptors (D1A, D1B, and D3) can be seen in the inner and outer halves of the molecular layer and in Purkinje cells, respectively (arrows). Scale bars = 0.25 mm in A (applies to A–F); 0.25 mm in G (applies to G–K); 0.25 mm in L.

Within the cerebellum, all receptors were expressed, but each had a specific pattern. All receptors were expressed in the granular (inner) layer, from low to high levels in the following order: D1A < D1B < D2 < D1D < D3 < D4 (Fig. 8A–F). In addition, D1A and D1B were expressed in complementary patterns in the inner and outer halves, respectively, of the molecular (outer) layer (Fig. 8G,H). D3 showed high expression in many of the Purkinje neurons and in the deep cerebellar nuclei (Fig. 8K,L).

For all brain regions studied, there were no noticeable expression differences between D2 receptor splice variants 1 (Figs. 4, 5, 7) and 5 (data not shown). For D3, we only tested the cDNA variant 1, because the other predicted variants by ENSEMBLE were not identified among our cDNA clones. There have been no predicted zebra finch variants for D4 to date.

**Figure 9:**
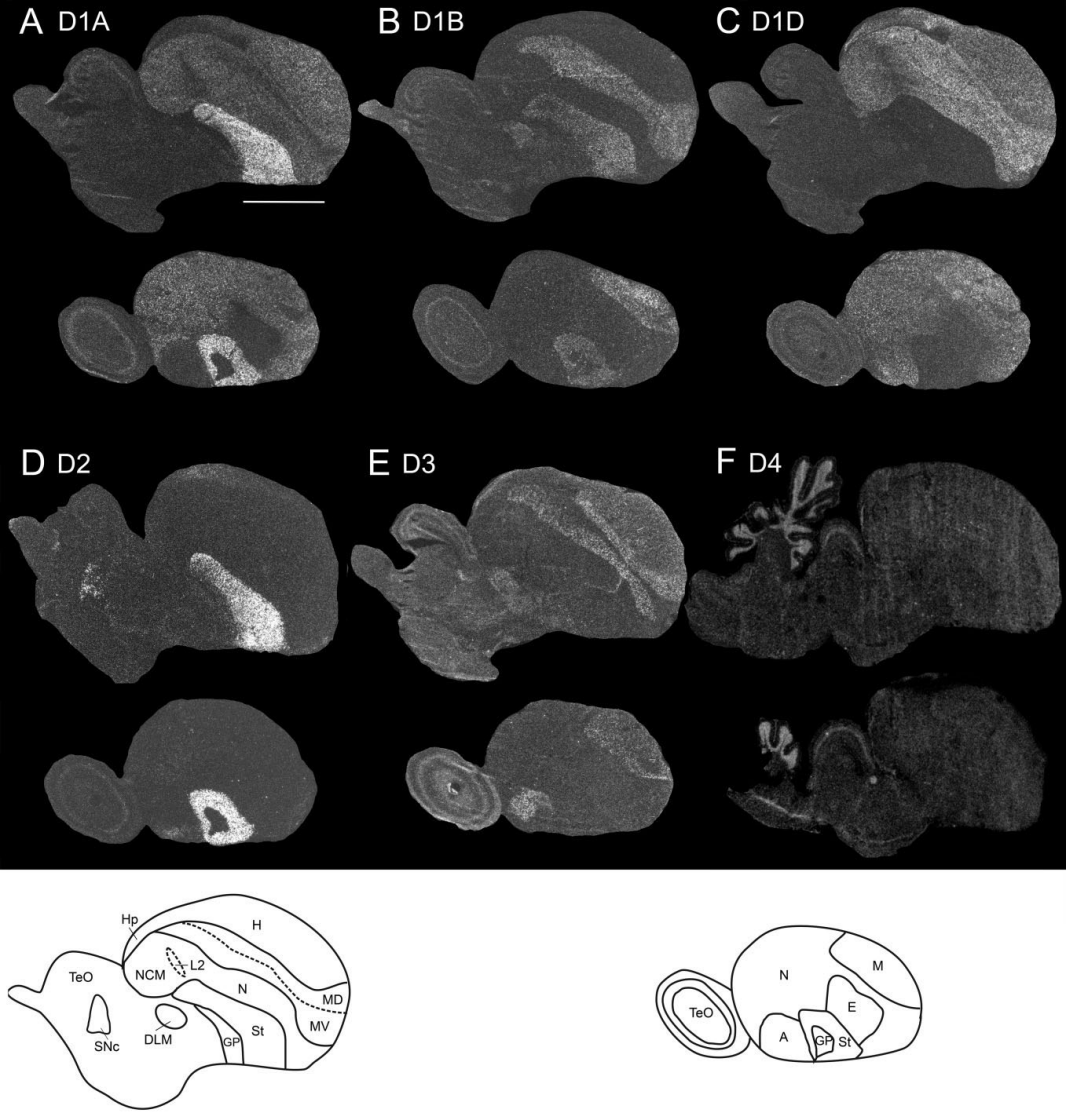
Expression profiles of dopamine receptor subtypes in adult chicken brain. Images were taken from film autoradiograms and inverted; white, mRNA signal. Two sagittal sections are shown per receptor; the distance from the midline is ∼1.5 (top) and 3.5 mm (bottom). A–C show the D1 receptor family (D1A, D1B, and D1D). D–F show the D2 receptor family (D2, D3, and D4). The hybridizations were done with the zebra finch ^35^S-UTP-labeled cRNA riboprobes using the same high-stringency conditions as for the zebra finch in situ hybridizations. Scale bar = 0.5 cm.

By using the zebra finch cDNAs as probes, we also performed a general expression analysis of these dopamine receptors in adult male chicken brains (Fig. 9). We found expression profiles similar to those of the zebra finch brain, but with some exceptions (compare Figs. 4 and 9). The most notable exception was expected, this being the absence of telencephalic song nuclei with differential expression (see below) in the chicken, a vocal nonlearner (Nottebohm and Nottebohm, 1971). Other differences were that, in the zebra finch, D1B is enriched at much higher levels in the striatum relative to the enriched expression in the mesopallium (Fig. 4), but, in the chicken, it is enriched at similar levels in the striatum and mesopallium (Fig. 9B), as previously reported (Sun and Reiner, 2000; Reiner et al., 2004a); D2 in the zebra finch is expressed at a detectable low, but enriched, level in the mesopallium relative to the remaining pallium (Fig. 4), but this enrichment is not seen in the chicken (Fig. 9D); D3 in the zebra finch is evenly enriched throughout the mesopallium (Fig. 4), but in the chicken it is unevenly enriched, with gradients of higher expression toward the boundaries of the dorsal and ventral mesopallium with the hyperpallium and nidopallium, respectively (Fig. 9E). The D1A, D1D, and D4 patterns are much more similar between zebra finch and chicken (Figs. 4, 6, 9A,C,F).

### Dopamine receptor expression in song nuclei

**Figure 10:**
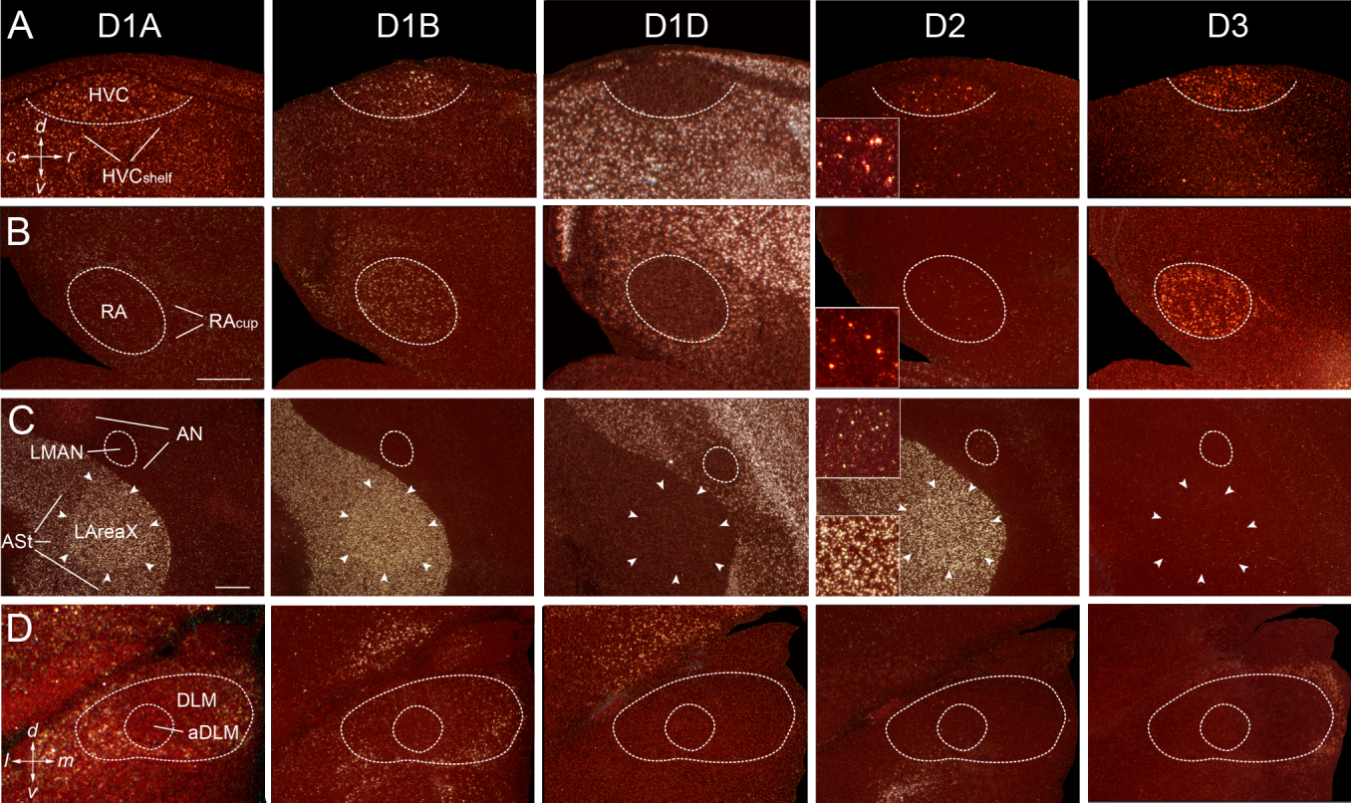
A–D: Higher power images of differential expression of different dopamine receptor subtypes in the song nuclei HVC, RA, LMAN, LAreaX, and aDLM of an adult male zebra finch. Insets for the D2 receptor show higher power images of isolated labeled cells in pallial song nuclei (HVC, RA, LMAN); inset for LArea X is shown for comparison. All sections are sagittal except for the last row, showing aDLM, which is coronal. White silver grains, dopamine receptor mRNA expression; red, cresyl violet stain. Scale bars = 0.5 mm (insets magnified ×2.7 more).

**Figure 11:**
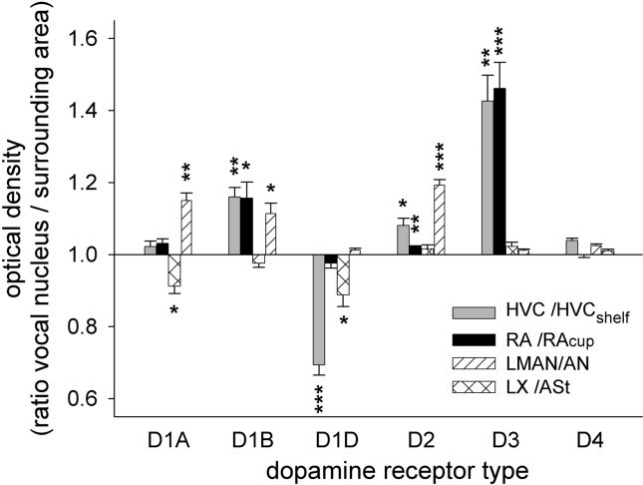
Quantification of the expression levels of the dopamine receptors in the song nuclei HVC, RA, LMAN, and LAreaX relative to their surrounding brain regions of HVC_shelf_, RA_cup_, AN, and ASt in adult male zebra finches. Each bar represents mean ± SEM measured from film autoradiograms. Statistical analysis was done by paired *t*-test where the ratio was compared with a ratio equal 1. *P < 0.05, **P < 0.01, ***P < 0.001.

Similarly to the specialized expression of glutamate neurotransmitter receptors in song nuclei (Wada et al., 2004), five of the six dopamine receptors showed differential expression in the song nuclei relative to the surrounding brain subdivisions. For the pallial song nuclei HVC and RA, four dopamine receptors showed differential expression; both nuclei had higher D1B, D2, and D3 expression and lower D1D expression relative to the surrounding nidopallium (HVC_shelf_) and arcopallium (RA_cup_), respectively (Figs. 10A,B, 11). However, the differential expression in RA for D1D and D2 was much less than that seen for HVC; for RA, we compared expression only with the surrounding intermediate arcopallium, because other subdivisions within the arcopallium showed differential expression relative to each other, and RA is located within the intermediate arcopallium (Reiner et al., 2004b). The differential D2 expression in both HVC and RA was due to high expression in isolated cells (Fig. 10A,B), indicating that differential expression could be cell type specific in these nuclei, such as in HVC's RA-projecting neurons, X-projecting neurons, or interneurons (Dutar et al., 1998; Kubota and Taniguchi, 1998; Mooney, 2000). LMAN showed generally low expression levels for all receptors, of which D1A and D1D were significantly lower than in the surrounding anterior nidopallium (AN; Figs. 10C, 11). For the D2 receptor, however, similarly to HVC and RA, there were isolated cells with higher expression levels in LMAN (Fig. 8C), but they were not numerous enough to be detectable as differentially expressed in the X-ray film quantifications. In Area X, all three striatally enriched receptors (D1A, D1B, and D2) were significantly expressed at higher levels above the surrounding anterior striatum (ASt; Figs. 10C, 11). D1D and D3 were low in Area X, similarly to the surrounding striatum. The D4 receptor showed no differential expression in any of the telencephalic song nuclei (Fig. 11), which is consistent with its barely detectable expression throughout the telencephalon (Fig. 6).

Within the thalamus, the anterior portion of DLM (aDLM), which we hypothesize to be the song nucleus part of DLM (Wada et al.,2004; Horita, Oka, Jarvis, Wada, in preparation), showed low to no detectable expression of dopamine receptors (Fig. 10D); there was lower D1A and D1B expression relative to that in the surrounding dorsal thalamus (Fig. 10D). In adult females, song nuclei are atrophied except for LMAN (Nottebohm and Arnold, 1976; Nixdorf-Bergweiler,2001), and there was a lower expression of D1D in “LMAN” relative to the surrounding AN (n = 3 females; data not shown). We did not note differential expression in any other areas of female brains where the song nuclei are located in males.

In summary, HVC, RA, and Area X expressed one or more dopamine receptors, which were higher than in the surrounding brain subdivisions. In contrast, LMAN and aDLM had low to no detectable expression of dopamine receptors, which in some cases was lower than that in the surrounding brain subdivisions. The D1D receptor was low if not absent in all of these nuclei relative to the rest of the telencephalon, and D4 was low or absent in both the song nuclei and the surrounding brain areas.

### Dopamine receptor expression in song nuclei during juvenile development

**Figure 12:**
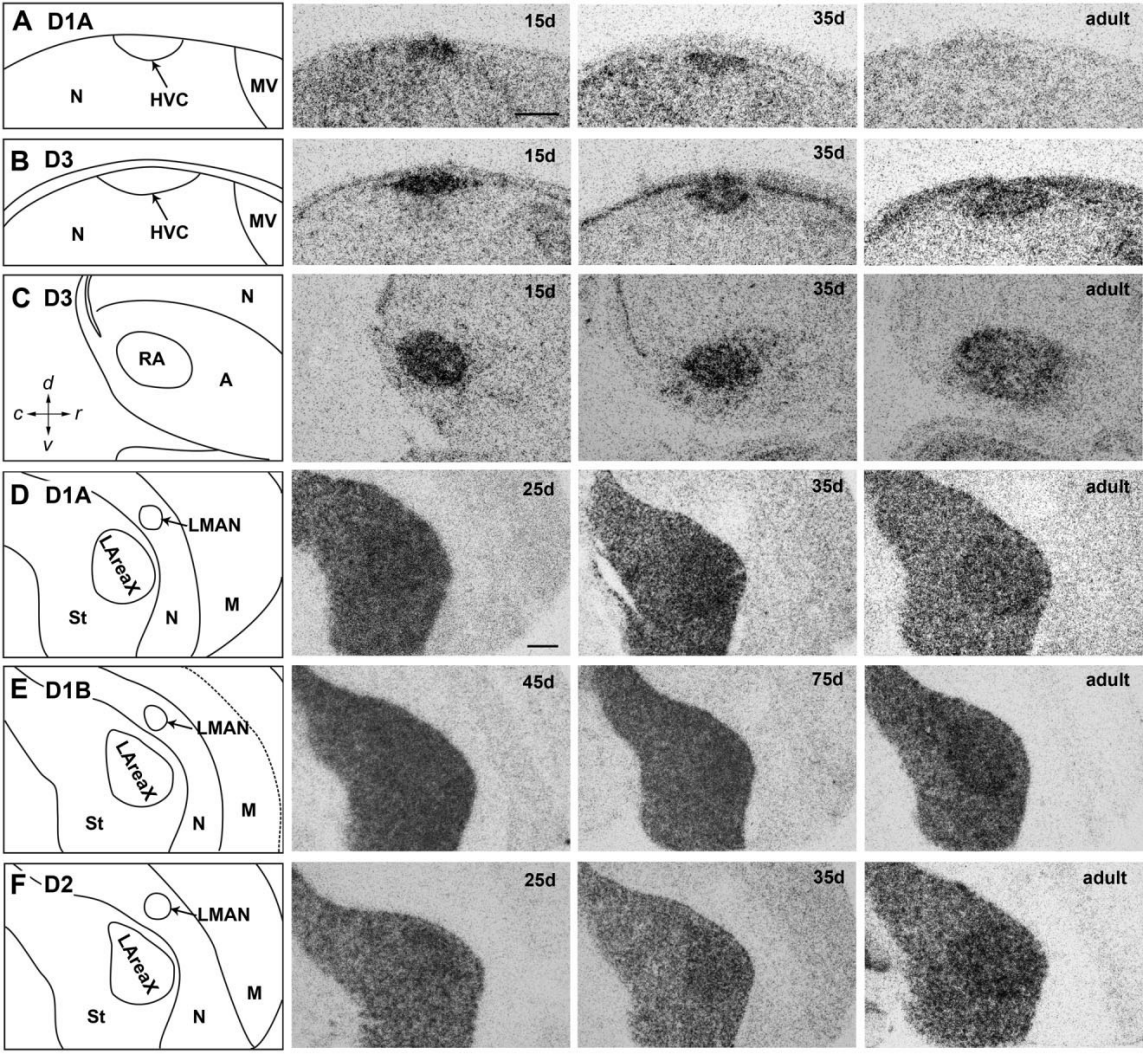
Images of differential dopamine receptor subtype expression in the song nuclei during development. A: D1A receptors in HVC. B: D3 in HVC. C: D3 in RA. D–F: D1A, D1B, and D2 in LArea X and LMAN, respectively. The age of each bird in days is labeled in the top right corner of each image. All sections are sagittal. The images were taken from film autoradiograms. Black, mRNA signal. Scale bars = 0.5 mm in A (applies to A–C); 0.5 mm in D (applies to D–F).

**Figure 13:**
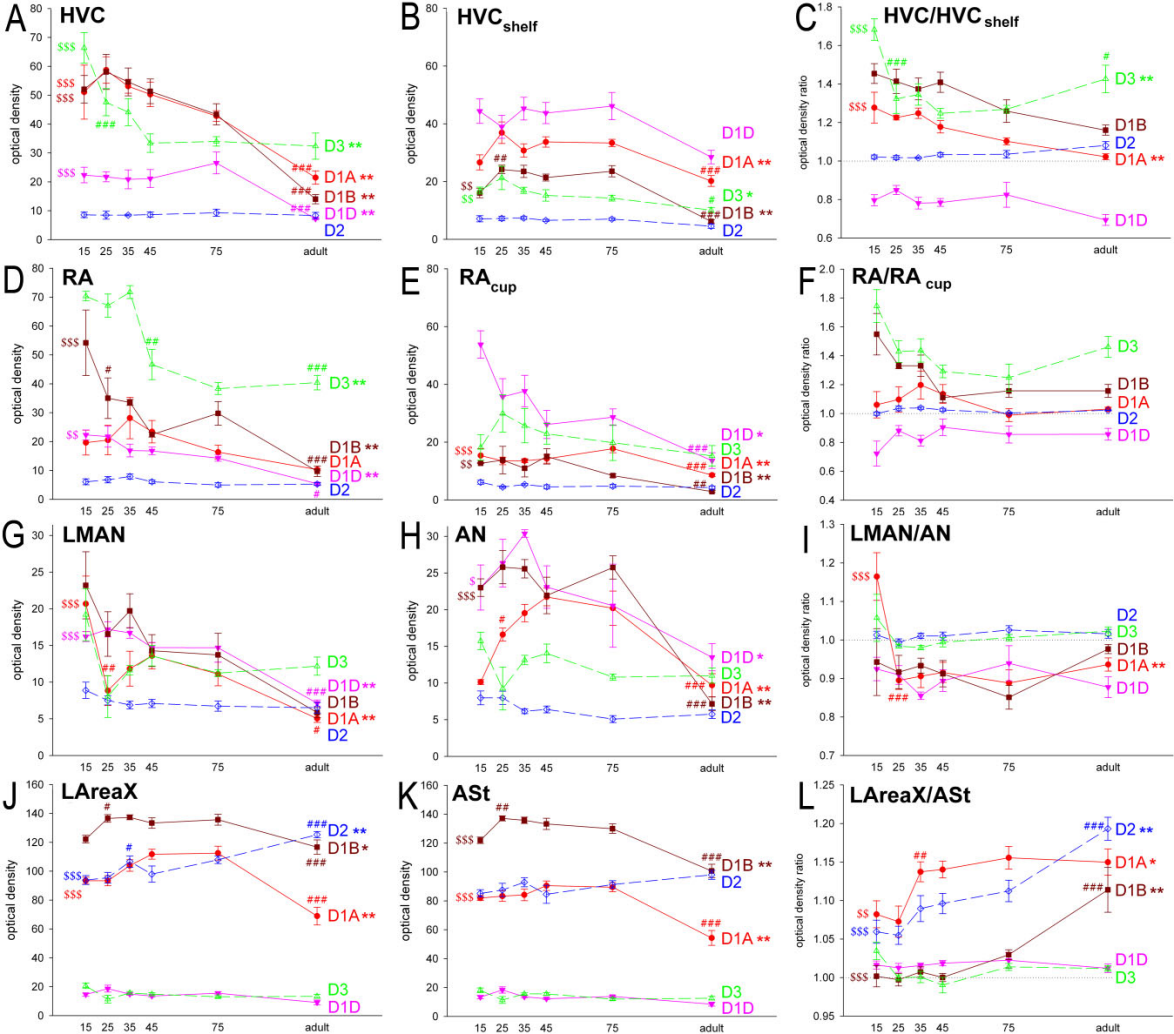
Quantification of dopamine receptor subtype expression in the song nuclei (A,D,G,J), surrounding areas (B,E,H,K), and their ratios (C,F,I,L) during development. Each point represents mean ± SEM. *P < 0.05, **P < 0.01, significant changes in densities during the whole of development assessed by ANOVA with Bonferroni correction. Fisher's PLSD post hoc tests revealed significant differences between the individual time points, but, for the sake of clarity, the figure shows only the differences between 15-day-old birds and adults as ^$$^*P* < 0.01 and ^$$$^*P* < 0.001 and differences between two adjacent time points (marked above the later one) as ^#^*P* < 0.05, ^##^*P* < 0.01, ^###^*P* < 0.001.

To quantify potential developmental differences in song nuclei, we performed two types of measurements: 1) analyses of absolute expression levels, which allowed comparisons among song nuclei and 2) analyses of relative expression levels to each immediate surrounding brain subdivision, which allowed assessment of specialized expression in song nuclei. Most dopamine receptors showed high absolute expression levels in song nuclei early in juvenile development (PHD15), followed by significant decreases in the pallial song nuclei during the sensory acquisition (PHD25–35), subsong (PHD35–45), plastic song (PHD75), and adult (>PHD90) crystallized song phases. Specifically, D1A, D1B, and D3 were higher in HVC during juvenile development and decreased in adulthood (Figs. 12A,B, 13A), whereas, in the surrounding HVC_shelf_, they initially increased, then remained stable during vocal development and finally decreased in adulthood (Fig. 13B). Despite the decreases in both HVC and HVC_shelf_, D1B and D3 expression still remained higher in HVC than in the surrounding nidopallium (i.e., HVC_shelf_) in adults (Figs. 11, 13C). Within RA, a similar developmental trajectory of decreased expression was found (Figs. 12C, 13D), except that D1A was not significantly higher than in surrounding intermediate arcopallium (RA_cup_) at any of the developmental ages studied (Fig. 13F). Within LMAN, all receptors, except for D2, were slightly higher in absolute levels in early juvenile development than in adulthood (Fig. 13G). The surrounding AN showed some increases, followed by decreases also for most receptors (Fig. 13H). However, relative to the surrounding AN, only D1A showed an abrupt change in LMAN, a sharp decrease between PHD15 and PHD25 (Fig. 13I). D4 in juvenile animals was still expressed at low levels throughout the telencephalon, with no apparent differences in song nuclei (Fig. 6).

Within Area X, among the three striatally expressed receptors, D1A and D1B showed initial increases followed by decreases in absolute expression levels during juvenile development, whereas D2 showed a gradual increase into adulthood (Fig. 13J). A similar result was found for the surrounding striatum (Fig. 13K). However, relative to the surrounding striatum, all three receptors showed gradual increases in Area X (Figs. 12D–F, 13L). The increased differential expression of D1A and D2 in Area X was already present at the sensory-motor phase of song learning (between days 25 and 35 for D1A, 25 and 45 for D2; *P* < 0.05) and increased further thereafter. The D1B differential increase occurred between the late plastic song phase (day 75) and adulthood (Figs. 12D–F, 13L). These findings suggest that D1A and D1B expression levels in the striatum surrounding Area X decreased more than they decreased in Area X, resulting in net higher levels in Area X in adulthood.

In summary, expression of most dopamine receptors in song nuclei and the surrounding brain subdivisions decreases during development. However, the decreases in relative expression are not uniform, such that expression in song nuclei can change independently of expression in the surrounding brain subdivisions.

### Colocalization of D1 and D2 receptors

**Figure 14:**
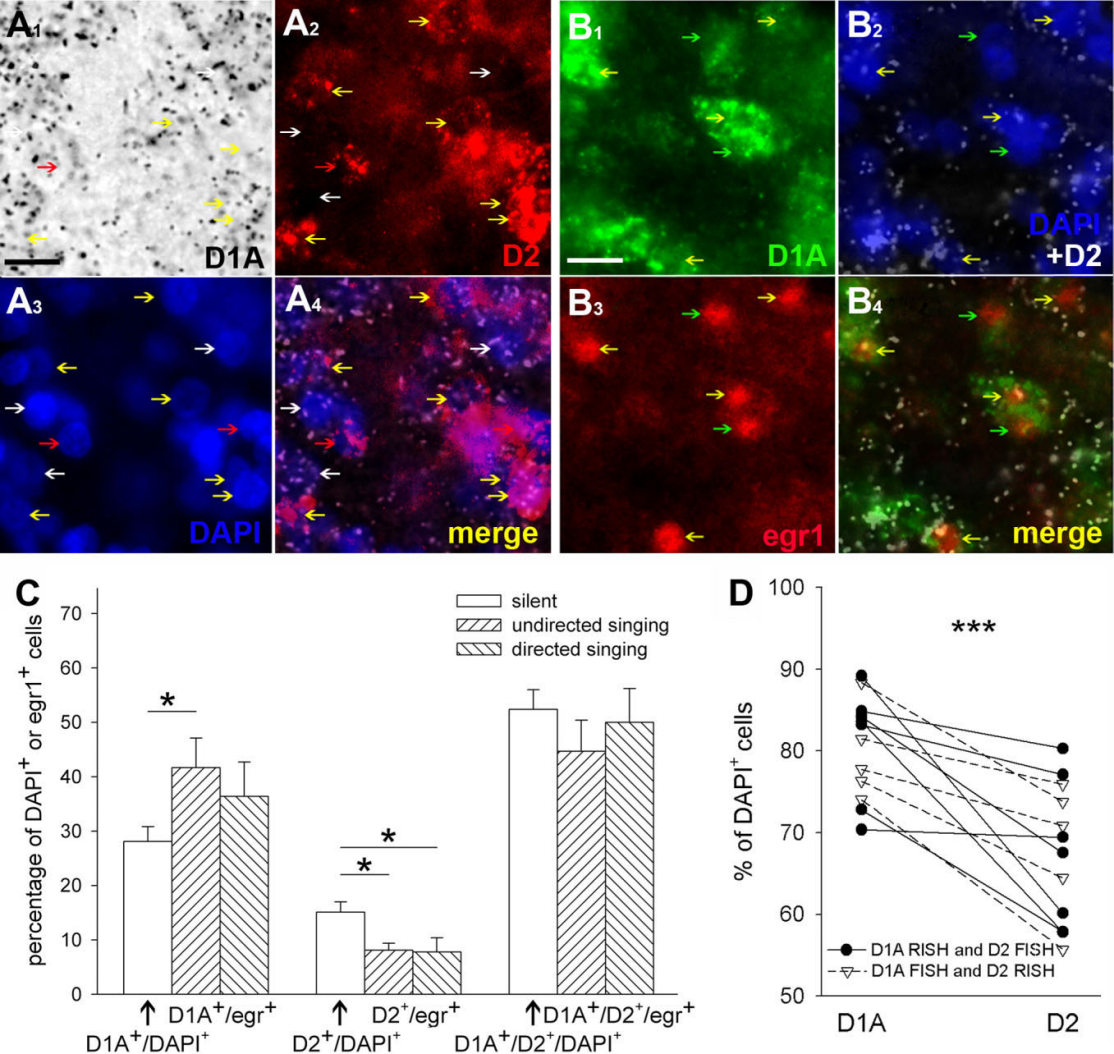
Images of D1A receptor, D2 receptor, and singing-driven egr1 colocalization in LArea X of the striatum. A1–A4: Colocalization of double-labeled D1A and D2 cells. A1, D1A receptor mRNA labeled with silver grains (black) in brightfield view using radioactive in situ hybridization (RISH); A2, D2 receptor mRNA labeled red using fluorescent situ hybridization (FISH); A3, cell nuclei labeled blue with DAPI; A4, merged image of D1A, D2, and DAPI; the D1A receptor signal is inverted and now silver grains are white. White arrows, D1A^+^/DAPI^+^ cells; red arrows, D2^+^/DAPI^+^ cells; yellow arrows, D1A^+^/D2^+^/DAPI^+^ cells. B1–B4: Colocalization of double-and triple-labeled neurons with D1A, D2, and undirected singing-driven egr1 expression. B1, D1A receptor mRNA in the cytoplasm labeled green using fluorescent in situ hybridization; B2, D2 receptor mRNA in the cytoplasm labeled with silver grains (white) above cell nuclei labeled blue by DAPI using radioactive in situ hybridization (image taken in darkfield view); B3, egr1 protein labeled red in the nucleus using immunocytochemistry; B4, merged image showing overlap of the D1A, D2, and/or egr1. Green arrows, D1A^+^/egr1^+^ neuron; yellow arrows, D1A^+^/D2^+^/egr1^+^ neuron. C: The proportion of cells expressing only D1A, only D2, or D1A and D2 receptors. Open bars show the proportion of cells for each receptor type relative to all cells (DAPI^+^, n = 1,226 across seven birds); hatched bars show the proportions relative to all egr1-labeled neurons after singing (after undirected singing, n = 747 egr1^+^ cells total or 124.5 ± 29.5 average egr1^+^ cells per bird, n = 6 birds; or after directed singing, n = 198 egr1^+^ cells total or 49.5 ± 13.2 average of egr1^+^ cells per bird, n = 4 birds). *P < 0.05, ANOVA followed by Fisher's PLSD post hoc test. D: Percentages of D1A-and D2-expressing cells comparing radioactive (RISH) and fluorescent (FISH) in situ hybridizations that were alternated for the D1A and D2 receptors. No differences in RISH vs. FISH results were found (*P* = 0.86 for D1A; *P* < 0.30 for D2 *t*-test, n = 6 animals, averaged over several sections). Regardless of probe combination, there is a higher percentage of D1A relative to D2-expressing cells (***P < 0.001, paired *t*-test between the percentages of D1A and D2 DAPI-labeled cells, single-or double-labeled, within LArea X of each animal). Scale bars = 10 μm.

Next we investigated whether D1A and D2 receptors exist in the same or in distinct cells of Area X. We chose to examine D1A and D2, because there has been a longstanding question for the mammalian striatum (for review see Callier et al., 2003) and recently for avian Area X (Ding and Perkel, 2002) regarding whether these two receptors are expressed in overlapping or distinct sets of striatal neurons. In double-labeling in situ hybridization experiments with one radioactive (RISH; D1A in Fig. 14A_1_, black silver grains) and one fluorescent (FISH; D2 in Fig. 14A_2_, red) probe, we found that in Area X at least 95% (Fig. 14C, total of silent group) of the cells (as detected by DAPI nuclear stain, Fig. 14A_3_, blue) expressed either D1A or D2 receptors [i.e., only 4.4% ± 0.7% (average ± SEM) were DAPI^+^ without D1A or D2 label]. More than half of the cells expressed both receptors simultaneously, i.e., were double labeled (Fig. 14C, D1A^+^/D2^+^/DAPI^+^). Among the single-labeled cells, the majority (28.1% ± 2.7% of the total cells) expressed the D1A receptor, whereas the minority (15.1% ± 1.9%) expressed the D2 receptor (Fig. 14C, D1A^+^/DAPI^+^ and D2^+^/DAPI^+^, respectively). When including all cells (single and double labeled), the majority still expressed D1A (80.5% ± 1.8%, average ± SEM) relative to D2 (67.6% ± 2.4%). This differential expression persisted also after alternating the probes (RISH vs. FISH, Fig. 14D), so these results were not influenced by the label combination used.

Because we noted that the contrast in the number of silver grains in labeled vs. unlabeled cells was stronger in single-label in situ hybridizations (compare Fig. 14A_1_,B_2_ with Supp. Info. Fig. 3A,B for D1A and D2, respectively), we counted the average numbers of D1A-and D2-containing cells there also. We found that 77.8% ± 2.4% (average ± SEM) of all Nissl-stained cells expressed the D1A receptor and that 70.2% ± 2.0% of all cells expressed the D2 receptor. These averages were not significantly different from the numbers of D1A (80.56% ± 1.86%; *P* = 0.36, *t*-test) and D2 (67.6% ± 2.46%; *P* = 0.44) DAPI-expressing cells quantified in the double-labeling experiment. Because the averages in single-label in situ hybridizations add up to 148.1%, these results indicate that there must be cells (at least 48.1%) that express both D1A and D2. Thus, the fluorescent + radioactive double-label and radioactive single-label in situ hybridizations yield similar conclusions.

We wanted further to determine whether the numbers of D1A^+^ and D2^+^ cells were similar or different in the surrounding ASt as in Area X. We found that, in ASt caudal-ventral adjacent to Area X, 72.7% ± 1.3% of all Nissl-stained cells expressed the D1A receptor and 66.4% ± 2.0% of all cells expressed the D2 receptor. This distribution in ASt did not differ significantly from the distribution in Area X (*P* = 0.26 for both receptors, *t*-test). However, the average number of labeled cells/×63 field was higher in Area X (average of 143.0 ± 9.4 for D1A and 131.1 ± 8.0 for D2) than in ASt (103.3 ± 8.8 for D1A and 101.3 ± 6.8 for D2; *P* < 0.05 for both, *t*-test). This result suggests that there is a higher density of cells (labeled and unlabelled) in Area X, which may explain in part its specialized expression.

In addition, we counted the numbers of D1B^+^ Nissl-stained cells in Area X and ASt. Remarkably, we found that D1B was expressed in 84.6% ± 1.5% of the Nissl-stained cells in Area X and 84.3% ± 1.8% in ASt. Similarly to the D1A and D2 receptors, the average number of labeled cells/×63 field was higher in area X (141 ± 4.9) than in ASt (105.7 ± 8.8; *P* < 0.05, *t*-test). Because the averages of D1B and D2 single-labeled cells add to 152.2%, these results predict that at least 52.2% cells in Area X would express both of these receptors.

### Social context, singing-induced egr1, and dopamine receptor colocalization

In mammals, D1 receptors up-regulate egr1 expression, whereas D2 receptors inhibit its expression (Gerfen et al., 1995). Given this knowledge, we hypothesized that, in songbirds, during undirected singing, the low levels of dopamine released by VTA-SNc axons onto Area X neurons would preferentially activate D1 receptors and thereby increase egr1 expression to high levels in Area X, whereas, during directed singing, the high levels of dopamine would preferentially activate D2 receptors and thereby dampen the singing-driven increase of egr1 expression in Area X (Sasaki et al.,2006; Hara et al., 2007). If this were true, one would expect a differential distribution of egr1 expression in D1-and D2-containing neurons during singing in different social contexts (egr1 is expressed only in neurons in an adult brain; Kubikova et al.,2007; Man et al., 2007). To address this hypothesis, we analyzed triple-labeling expression to colocalize D1A (Fig. 14B_1_, green) and D2 (Fig. 14B_2_, white silver grains) receptor mRNA with singing-induced egr1 protein (Fig. 14B_3_, red; merged in Fig. 14B_4_) in lateral Area X (LArea X), the portion of area X that shows social-context-dependent gene regulation (Jarvis et al.,1998).

We found that, after undirected singing, the majority (86.4% ± 2.3%, average ± SEM) of the egr1^+^ neurons were D1A^+^ and D1A^+^/D2^+^ (Fig. 14B_4_,C, D1A^+^/egr1^+^, D1A^+^/D2^+^/egr1^+^); a small minority were D2^+^ only (8.1% ± 1.3%; Fig. 14C, D2^+^/egr1^+^) or did not express either dopamine receptor (5.4% ± 1.2%, not shown in graph). After directed singing, the average number of egr1^+^ neurons was less than that after undirected singing (33 ± 11.5 for directed vs. 93.4 ± 9.0 for undirected egr1^+^ cells per section, average ± SEM), as expected (Jarvis et al., 1998). The distribution of egr1 expression among D1A^+^, D2^+^, and D1A^+^/D2^+^ neurons during directed singing was similar to that seen during undirected singing (Fig. 14C). However, when we compared the distribution of D1A and D2 labeled cells colocalized with egr1^+^ to the distribution of all D1A and D2 labeled cells (colocalized with DAPI), we found significant differences across singing contexts. During undirected singing, there was a significant 148% overrepresentation of D1A^+^ neurons (Fig. 14C, D1A^+^/egr1^+^) and a 54% underrepresentation of D2^+^ neurons with egr1 expression (Fig. 14C, D2^+^/egr1^+^). During directed singing, there was a significant 52% underrepresentation of D2^+^ neurons with egr1 expression (Fig. 14C, D2^+^/egr1^+^). In summary, these findings suggest that during undirected singing egr1 is preferentially expressed in D1A neurons and preferentially not expressed in D2 neurons and that during directed singing egr1 is preferentially not expressed in D2 neurons.

## DISCUSSION

The dopaminergic system in vertebrates has been studied for its function in multiple behaviors, such as motor activity, cognition, motivation, reward, learning (including learned vocal communication), sleep, and mood (Salamone, 1992; Pace-Schott and Hobson,2002; Wise, 2004; Doupe et al., 2005; Panksepp, 2005; Berridge, 2007; Schultz, 2007). However, little knowledge exists on the function of dopamine receptors in the central nervous systems of birds. In this study, we cloned cDNAs of all six dopamine receptors, D1A, D1B, D1D, D2, D3, and D4, from the avian brain. We determined their phylogenetic relationships and brain distributions in adult zebra finches (a songbird) and chickens (galliformes), in juvenile zebra finches during different song learning phases, and in zebra finches during singing in different social context. Below we discuss the evolution of dopamine receptors, their localization, their significance for vocal learning, and the implications for social-context-dependent singing behavior.

### Evolution of dopamine receptors in birds

Our sequence analyses suggest that, as in mammals (Callier et al., 2003), the D1 family of receptors in birds exists as single exon genes, and the D2 family exists as spliced multiple exons (for chicken see Demchyshyn et al., 1995). This finding and the fact that the D1 and D2 receptor protein sequences are more related to other monoamine receptors (serotonin, adrenergic, and trace amine receptors) than they are to each other support the conclusion that the ancestral D1 and D2 receptors diverged from duplications of a seven-transmembrane receptor, had two separate evolutionary histories, and then converged on their ability to bind dopamine independently (Callier et al., 2003). After convergence, further gene duplication apparently led to the different receptor subtypes of each family among vertebrates. It is believed that D1C was subsequently lost in mammals and D1D subsequently was lost in most vertebrates except for birds, or that D1D evolved independently in birds (Demchyshyn et al., 1995; Callier et al., 2003; Le Crom et al., 2004). Our sequence analyses suggest a distant relationship of avian D1D to D1A and D1B, supporting the possibility that D1D was lost in other vertebrates (Callier et al., 2003).

Some chicken sequences in the public databases (NCBI) have been annotated as D1C. However, we find that these sequences are 100% identical to chicken D1D (Demchyshyn et al.,1995) and share close identity to our zebra finch D1D clone. Furthermore, we have not been able to find a separate D1C-like sequence in the currently sequenced zebra finch or chicken genomes (as determined by BLAT searches with *Xenopus* D1C). This suggests that D1C might have been lost in both mammals and birds. Determining whether it was lost before the ancestral split between birds and mammals requires determining whether D1C is present in nonavian reptiles. We have not been able to find D1C sequences in 454 high-throughput cDNA sequences of crocodile brain (unpublished findings). We also could not find any dopamine receptors in the current draft of the zebra finch genome other than the six we describe here. Given these findings, we might have identified all dopamine receptors in the songbird, and they do not include D1C. An alternative interpretation is that avian D1D and fish/frog D1C are really homologs, but one or both of them underwent rapid divergence resulting in lower sequence identities than expected. This later idea is supported by a recent preliminary report (Yamamoto and Vernier 2010 Soc. for Neurosci. Abs 23.1) that showed avian D1D and fish and frog D1C have chromosomal synteny of genes surrounding them, suggesting that they are homologs.

For the D2 receptor family, our analyses suggest that there are multiple spliced forms that affect the third cytoplasmic loop. This loop is important for intracellular signaling functions of dopamine receptors. The loop physically links the G-protein component of the receptor to cAMP, adenylate cyclase, and extracellular kinase (ERK) proteins, which in turn regulate downstream processes, including modulation of activity-dependent gene expression such as egr1 (Ilani et al., 2002; Takeuchi and Fukunaga, 2004). We have not seen a brain expression difference between the two D2 zebra finch cDNA splice variants 1 and 5 thus far discovered, indicating that the different functional proteins could work in the same brain regions, if not the same neurons. However, some differential expression of D2 splice variants of the third cytoplasmic loop have been found in mammals by RT-PCR, in which the longer variant (variant 1) is expressed at slightly higher levels in the striatum and the shorter one at higher levels in the VTA-SNc (Guivarc'h et al., 1995; Khan et al., 1998). It is possible that these small differences are not easily detected by in situ hybridization. In general, the variable regions of homology that we found between zebra finch and human vs. chicken and human for the D2, D3, and D4 receptors suggest that the D2 family of receptors may be rapidly evolving and that these changes occurred after galliformes and songbirds split from their common ancestor.

### Brain expression profiles of dopamine receptors

The most salient finding of our expression analysis is that the more canonical and apparently ancestral dopamine receptors (D1A, D1B, and D2) are enriched in the striatum, which is innervated by VTA-SNc, whereas the more derived receptors (D1D and D3) are enriched in pallial regions, which is innervated by other midbrain dopaminergic and noradredergic neuron groups (e.g., GCt and LoC). These brain expression patterns appear to be representative of birds, insofar as they were similar in zebra finch (except song nuclei) and chicken, species that are distantly related to each other (Hackett et al., 2008). The patterns we found are consistent with previous reports of D1A and D1B mRNA expression patterns in chicken (Sun and Reiner, 2000); D1-like and D2-like receptor ligand binding patterns in chickens, quails, and pigeons (Richfield et al., 1987; Dietl and Palacios, 1988; Ball et al., 1995; Schnabel and Braun, 1996; Stewart et al., 1996; Schnabel et al.,1997; Kostal et al., 1999); and the D1-like receptor binding pattern in starling (Casto and Ball, 1994; Heimovics et al.,2009). The latter studies are the only previous distribution studies of dopamine receptors in a songbird. The most recent (Heimovics et al., 2009) found relationships between D1-like densities in midbrain dopaminergic neurons and breeding context. We found some species differences in the pallium between zebra finches and chickens, most notably in the mesopallium, where D1B was higher, D2 lower, and D3 expressed in gradients in chicken relative to zebra finch. This suggests that, for dopamine receptor gene regulation in the mesopallium, there could be cis-regulatory mutations between zebra finch and chicken to control species-specific differential expression.

In contrast to the conserved pattern that we found in zebra finch and chicken, there is a substantial discrepancy with the D2 mRNA pattern previously reported in turkey brain (Schnell et al.,1999). Although we found that D2 mRNA was highest in the striatum of both zebra finch and chicken, Schnell et al. ( 1999) found that it was nearly all pallial in turkey. To explain a pallial mRNA pattern that does not correlate with the D2 ligand binding pattern in birds, Schnell et al. ( 1999) proposed that avian D2 mRNA may be transported to the striatum and synthesized into protein, and then the mRNA rapidly degraded in the striatum. We believe that this hypothesis is unlikely in light of our findings; it would mean that D2 mRNA synthesis, transport, and degradation are all very different in two very closely related species, chicken and turkey, but not different in two distantly related species, chicken and zebra finch. Thus, we wondered whether the turkey result represented differential expression of D2 splice variants, but we find that the authors' turkey cDNA probe (accession No. AF056201) is the homologue to the chicken D2 cDNA variant 1 (96%; No. EU313425 by Zu and Hang) and our zebra finch D2 cDNA variant 1 (86%; Supp. Info. Fig 2B). Thus, we believe that either the turkey is different from other avian species or the difference between studies is due to an unknown technical factor. In either case, our results suggest that D2 is highly expressed in the avian striatum across species, as in mammals.

The avian dopamine receptor patterns we found are consistent with the new view of avian brain organization and homologies with mammals and other vertebrates (Reiner et al., 2004a; Jarvis et al., 2005). As in birds, the D1A, D1B, and D2 receptors are most abundant in the mammalian striatum and proposed fish striatum, relative to much lower levels of isolated expression in pallial areas (Meador-Woodruff et al., 1991; Weiner et al., 1991; Rappaport et al., 1993; Choi et al., 1995; Kapsimali et al., 2000; Hurd et al., 2001). Furthermore, as in birds, the D2 receptor in mammals is specifically enriched in the VTA-SNc (Vallone et al., 2000; Callier et al., 2003). As in birds, the D3 receptor in mammals is expressed at lower levels in the brain, with expression restricted mainly to pallial regions, including a sparse distribution in the hippocampus and higher expression in layers V–VI of anterior cortical regions (possibly analogous cell type to avian arcopallium projection neurons; Jarvis et al., 2005) and in Purkinje neurons of the cerebellum (Bouthenet et al., 1991; Landwehrmeyer et al.,1993a,b; Diaz et al., 1997). Although there is high D3 (and D1B) in the avian mesopallium, there is no agreement on a homologous structure or cell group in mammals (Jarvis et al., 2005). For the D4 receptor, large discrepancies exist regarding where it is expressed in the mammalian brain (Noain et al., 2006); these discrepancies are thought to be due to use of nonspecific antibodies and ligands. With a sensitive BAC transgene in front of the D4 promoter, similar to the case in zebra finch, D4 was found to be low to absent throughout most of the mouse telencephalon (Noain et al., 2006). However, high expression was found in discrete isolated cells of the deep layers of the prefrontal cortex and of the ventral pallidum (Noain et al., 2006). Unlike the case in the zebra finch brain, no D4 transgene expression was found in the mouse cerebellum, although prior studies reported D4 protein expression in the rat cerebellum with antibodies (Barili et al., 2000). In both mammals and chicken, D4 is expressed at high levels in the retina (Rohrer and Stell, 1995; Zawilska et al., 2003).

The low forebrain expression levels of the D4 receptor are intriguing given prior findings of its role in novelty behavior and attention deficit hyperactivity disorder (ADHD). Deletions, inserts, and single nucleotide polymorphisms of the D4 receptor gene are all associated with variations in novelty-seeking behavior, ADHD, and other personality traits in the great tit (a songbird), humans, vervet monkeys, and horses (Kluger et al., 2002; Momozawa et al., 2005; Bailey et al., 2007; Fidler et al., 2007). A subset of humans with ADHD and an associated repetitive sequence mutation in their D4 receptor protein sequence have relatively smaller volumes of prefrontal cortex and cerebellar cortex. Insofar as ADHD, novelty seeking, and other behaviors presumably involve the telencephalon, it is possible that low, but barely detectable, levels of the D4 receptor in the telencephalon lead to robust functions upon binding dopamine.

The different dopamine receptors have different binding affinities for dopamine and different response after binding dopamine. The D2 receptors have the highest binding affinities, followed by different D1 affinities in the order D3 > D4 > D2 > D1D > D1B > D1A; each receptor also has different agonist-dependent desensitization, e.g., D1A > D1B (Demchyshyn et al., 1995; Kostal et al., 1999; Vallone et al.,2000; Callier et al., 2003; Kubikova et al., 2009). The pharmacological binding profile of the dopamine receptors to various agonists and antagonists is very similar among the avian, mammalian, and other vertebrate species (Demchyshyn et al., 1995; Kubikova et al., 2009). This suggests that, after dopamine release and binding, specific brain areas and neuron types will have functional differences depending on densities of the individual receptor types.

A longstanding issue with regard to dopamine receptors in the mammalian brain is whether D1 and D2 receptors are expressed in the same striatal neurons or in different ones. Some studies report segregation into distinct neuronal populations (Gerfen et al., 1990; Le Moine and Bloch, 1995), whereas other studies report coexpression in a moderate number to nearly all striatal spiny neurons (Ariano et al., 1997; Aizman et al., 2000; Lee et al., 2004). Differences between studies could be due to differences in techniques, differences between receptor subtypes examined (D1A vs. D1B), or species differences. In songbirds, a previous study using pharmacological manipulations suggested that there is considerable (qualitative) overlap of D1-like and D2-like receptor physiological responses in the same neurons of Area X, but also some segregation, in which the D1-like receptors mediated enhancement of excitability at a hyperpolarized state and the D2-like receptors mediated its reduction (Ding and Perkel, 2002). Consistently with these findings, we show that, in LArea X, about half of the cells express mRNAs for both D1A and D2 receptors. Other cells contained either D1A or D2 receptors, but the number of D1A-only cells was twice as high as the number of D2-only cells. We believe that a similar distribution will apply to the remaining striatum, insofar as we did not note visibly gross differences in the distribution of double labeling outside of LArea X, and the proportion of single-labeled cells in ASt was similar to that in LArea X. It is not clear, however, whether the mRNA proportions are translated one-to-one into receptor protein densities. The ligand binding studies do not help us to answer this question, because there is no consensus on whether there are more D1-like receptors (Schnabel and Braun, 1996; Kleitz et al., 2009), comparable amounts of D1-like and D2-like receptors (Stewart et al., 1996; Kostal et al., 1999), or more D2-like receptors (Richfield et al., 1987; Stewart et al., 1996) in the avian striatum. With these ligand studies, it is not possible to determine the receptor types. It is possible that some proportion of cells contain not only D1A^+^/D2^+^-expressing neurons but D1B^+^/D2^+^ expression neurons, as suggested from quantifications of our single-labeling in situ hybridizations. Although we have not been able to find another study that examined whether D1B and D2 are coexpressed in the same neurons in intact brain, in transfected cells these receptors are able to form heterooligomers (So et al., 2009).

### Possible functional consequences for vocal learning

We found that, during all stages of song development, there is a large overlap in expression of genes belonging to the D1 and D2 receptor families in three song nuclei (HVC, RA, and LAreaX), but with differential abundance that presumably results in different functional dopamine responses of each song nucleus. In LMAN, there are relatively low levels of dopamine receptors regardless of the developmental stage. This finding suggests a lesser function of dopamine in LMAN.

The developmental changes of dopamine receptor levels in song nuclei roughly correspond to their dopamine and dopamine turnover levels and inversely correlate with tyrosine hydroxylase immunoreactivity levels (Soha et al., 1996; Harding et al., 1998); tryrosine hydroxylase is the rate-limiting enzyme for dopamine synthesis. Unlike the receptor levels determined in this study, dopamine levels are low in song nuclei in young, ∼20-day-old, birds and increase to higher levels in birds between 35 and 55 days old, but, thereafter, as the birds become adults, the dopamine levels, like the receptors, decrease (Harding et al., 1998). Inversely to the receptors, the density of tyrosine hydroxylase fibers in song nuclei gradually increases relative to the surrounding brain regions throughout juvenile development (Soha et al., 1996). For the receptors, we further found that some changes (increase or decrease) in song nuclei are coregulated with changes in the surrounding brain subdivisions, but not by as much. Other changes appear to be specific to the song nuclei; these are the decreases of D1A, D1B, and D3 receptors in HVC and of D1B and D3 in RA. Taken together, these findings suggest that, although there is decreased dopamine receptor expression and dopamine levels in song nuclei during development, the decrease is not as strong as in the surrounding brain areas, and it is countered by increased tyrosine hydroxylase fiber innervation and thus presumably dopaminergic innervation from the midbrain.

The differential expression of dopamine receptors in song nuclei from juvenile development to adulthood could have important consequences for song learning and song production. The higher levels of D1B and D3 receptors in adult HVC and RA could lead to differential dopamine control of motor circuits for singing relative to motor circuits adjacent to song nuclei that control other behaviors (Feenders et al., 2008). The increases in D1A receptor levels in Area X between days 25 and 35, and in D2 receptor levels between days 25 and 45, could lead to higher activation of Area X neurons (D1A) in the sensory acquisition phase, followed by counteraction of this response (D2) in the plastic subsong phase of song learning. This hypothesis is testable and perhaps plausible given the known role of dopamine in learning in mammals (Reynolds and Wickens, 2002; Schultz, 2007) and birds (Stewart et al., 1996; Rose and Stewart, 1999). In young chickens, 30 minutes after one trial of passive avoidance training, there is a large increase of D1-like but not D2-like receptor binding in the medial striatum (Stewart et al., 1996; terminology adopted from Reiner et al., 2004a), and this is accompanied by increases in the size of the synaptic density in the same structure (Rose and Stewart, 1999). In songbirds, systemic administration of D1/D2 antagonist between posthatch days 45 and 57 decreases directed singing in adult zebra finch (Harding, 2004), but the authors did not study song learning.

### Dopamine receptors in Area X and singing-related activation

A frequently asked question is what is the mechanism of social-context-dependent activation in LArea X during singing, where there are high levels of egr1 mRNA and protein, and neural activity during undirected singing and low levels during directed singing (Jarvis et al., 1998; Hessler and Doupe, 1999; Kubikova et al., 2007). Plausible candidates for this modulation are the dopamine receptors, insofar as dopamine is present in LArea X at high levels during directed singing and at low levels during undirected singing (Sasaki et al., 2006) and dopamine is released from the midbrain cell groups VTA and SNc (Lewis, 1981; Gale and Perkel, 2006; Castelino et al., 2007; Hara et al., 2007; Gale et al., 2008; Person et al., 2008), which also exhibit social-context-dependent differences in activity and gene regulation (Yanagihara and Hessler, 2006; Hara et al., 2007).

Our results suggest that, during undirected singing, egr1 expression in LArea X occurs mainly in neurons containing D1A receptors with or without D2 receptors and less so in neurons that contain only D2 receptors. Such an effect is consistent with findings in the mammalian striatum, where, when acting alone, activation of D1-like receptors increases and activation of D2-like receptors decreases egr1 expression. Our findings are also consistent with electrophysiological actions of both receptors in birds and mammals, in which D1-like receptors excite and D2-like receptors inhibit evoked activity in mammalian striatum and in area X neurons (Ding and Perkel, 2002). The smaller number of egr1^+^ neurons after directed singing could be a consequence of a dominant function of D2 receptors in D1A^+^/D2^+^ cells with high dopamine levels. However, it has been found that synergistic activation of D1-like and D2-like receptors in the same neurons can lead to even greater increases in egr1 expression than the activation of D1-like receptors alone (Keefe and Gerfen, 1995; Wirtshafter, 2007). If this were to occur in intact brain, then an alternative mechanisms could be responsible for the social context differences, such as the affinity state of D2 receptors and the amount of dopamine release (Richfield et al., 1989). Finally, other systems might play a role in modulating social-context-dependent egr1 expression. For example, depletion of noradrenergic input leads to increased egr1 expression in Area X after directed singing (Castelino and Ball, 2005), and dopamine has been shown to bind to alpha-2-adrengeric receptors in Area X (Cornil et al., 2008), suggesting the involvement of the noradrenergic system in regulation of social-context-dependent activation of Area X. In summary, this study provides a basis for deciphering dopamine function in the avian brain, differential dopamine activation of different avian brain subdivisions, and regulation of dopamine in song nuclei during song learning and social-context-dependent singing.

## Abbreviations

A: arcopallium
aDLM: anterior nucleus of DLM
AN: anterior nidopallium
APH: area parahippocampalis
Area X: Area X of the striatum
ASt: anterior striatum
B: basolateralis
Cb: cerebellum
CMM: caudomedial mesopallium
CO: optic chiasma
DLA: dorsolateral anterior nucleus of the thalamus
DLM: dorsal lateral nucleus of dorsomedial thalamus
DM: dorsal medial nucleus of the midbrain
DMA: dorsomedial anterior nucleus of the thalamus
DMP: dorsomedial posterior nucleus of the thalamus
DNH: dorsal nucleus of the hyperpallium
E: entopallium
GCt: mesencephalic central gray
GP: globus pallidus
H: hyperpallium
Hp: hippocampus
HVC: nucleus HVC, a letter-based name
IH: interstitial layer of the hyperpallium
IPC: nucleus isthmi, pars parvocellularis
LArea X: lateral Area X
LMAN: lateral magnocellular nucleus of anterior nidopallium
L2: Field L2
M: mesopallium
MArea X: X medial Area X
MD: dorsal mesopallium
MLd: dorsal part of the lateral mesencephalic nucleus
MMAN: medial magnocellular nucleus of anterior nidopallium
MSt: medial striatum
MV: ventral mesopallium
N: nidopallium
NCM: caudal medial nidopallium
NIf: nucleus interface of the nidopallium
NIII: occulomotor nerve III
nXIIts: tracheosyringeal part of the hypoglossal nerve
PT: pretectal nucleus
RA: robust nucleus of the arcopallium
RAm: nucleus retroambiguus
Rot: nucleus rotundus
SNc: substantia nigra, pars compacta
St: striatum
SVZ: subventricular zone
TeO: optic tectum
Uva: nucleus uvaeformis
V: ventricle
VP: ventral pallidum
VTA: ventral tegmental area

## Supplementary material

Fig. S1 Comparative protein sequence analyses of zebra finch dopamine receptor subtypes relative to chickens and humans. A-F: Receptor alignments for D1A, D1B, D1D, D2, D3, and D4, respectively. The protein coding sequences were aligned with clustalW www.ebi.ac.uk/Tools/clustalw2/ and then manually edited to correct errors in alignments. Text color coding: Red, small+hydrophobic, including aromatic (-Y) amino acids; Blue, acidic a.a.; Magenta, basic a.a.; Green, hydroxy+amine+basic (-Q) a.a.. Receptor domains were predicted with TMHMM software ( www.cbs.dtu.dk/services/TMHMM/) based on the zebra finch sequences. The exact predictions can slightly differ for different species, based on sequence differences. TM, transmembrane domain (boxed regions); CL, cytoplasmic loop; EL, extracellular loop. Outside and inside domains are the amino-and carboxy-terminal regions, respectively, that are positioned outside and inside of the cell, respectively. Note that the TM domains are mainly hydrophobic. For D1B (B), the first ∼23 a.a. of the zebra finch protein has not yet been sequenced from the zebra finch genome or cloned as a cDNA, and thus the sequence shown is partial. For D1D (C), the chicken sequence shown is the one annotated by some sources as D1C. For D2 (D), variant 1 for birds and the variant long of humans are aligned. For D3 (E), the prediction algorithms generated a longer protein at the amino terminal end in chicken than that supported by avian EST evidence and homologies to zebra finch and other vertebrate species (our analysis). Thus, we truncated the chicken sequence at the start site for zebra finch. The closest human D3 variant (variant 1) to the zebra finch protein was aligned. For D4, the zebra finch sequence between the arrows was determined from the cDNA clone of this study (part of which has not yet been sequenced yet in the genome), whereas the remaining sequence was determined from the genome. Accession numbers of the clones used are shown in Fig. 2.

Fig. S2 Zebra finch D2 receptor variant alignments. A: Protein sequence alignments of cDNA supported and genomic predicted protein sequences of D2 splice variants. The cDNA inferred protein variants 1 and 5 (D2v1 and D2v5) were cloned in this study. The variants 3-4 (D2v1-D2v4) were predicted by ENSEMBLE and are in NCBI Genbank. Color-coding and labels follow the format described in the legend of Fig. S1. Note the splice variations in the 3rd cytoplasmic loop (CL3). B: Alignments of the zebra finch D2 variant 1 used for in situ hybridizations in this study with chicken D2 variant 1 and the turkey D2 long variant used by Schnell et al (1999).

Fig. S3 Images from single label radioactive in-situ hybridization showing A: D1A and B: D2 receptor mRNA (silver grains in emulsion; black dots) above Nissl labeled cells (grey) in Area X of the striatum in zebra finch. Arrows, labeled cells; arrow heads, non-labeled cells. Scale bar, 10 μm.

## References

[b1] Aizman O, Brismar H, Uhlen P, Zettergren E, Levey AI, Forssberg H, Greengard P, Aperia A (2000). Anatomical and physiological evidence for D1 and D2 dopamine
receptor colocalization in neostriatal neurons. Nat Neurosci.

[b2] Appeltants D, Absil P, Balthazart J, Ball GF (2000). Identification of the origin of catecholaminergic inputs to HVc
in canaries by retrograde tract tracing combined with tyrosine hydroxylase
immunocytochemistry. J Chem Neuroanat.

[b3] Ariano MA, Larson ER, Noblett KL, Sibley DR, Levine MS (1997). Coexpression of striatal dopamine receptor subtypes and
excitatory amino acid subunits. Synapse.

[b4] Aronov D, Andalman AS, Fee MS (2008). A specialized forebrain circuit for vocal babbling in the
juvenile songbird. Science.

[b5] Bailey JN, Breidenthal SE, Jorgensen MJ, McCracken JT, Fairbanks LA (2007). The association of DRD4 and novelty seeking is found in a
nonhuman primate model. Psychiatr Genet.

[b6] Ball GF, Casto JM, Balthazart J (1995). Autoradiographic localization of D1-like dopamine receptors in
the forebrain of male and female Japanese quail and their relationship with
immunoreactive tyrosine hydroxylase. J Chem Neuroanat.

[b7] Barclay SR, Harding CF (1988). Androstenedione modulation of monoamine levels and turnover in
hypothalamic and vocal control nuclei in the male zebra finch: steroid
effects on brain monoamines. Brain Res.

[b8] Barclay SR, Harding CF, Waterman SA (1992). Correlations between catecholamine levels and sexual behavior in
male zebra finches. Pharmacol Biochem Behav.

[b9] Barili P, Bronzetti E, Ricci A, Zaccheo D, Amenta F (2000). Microanatomical localization of dopamine receptor protein
immunoreactivity in the rat cerebellar cortex. Brain Res.

[b10] Berridge K (2007). The debate over dopamine's role in reward: the case for
incentive salience. Psychopharmacology.

[b11] Bottjer SW (1987). Ontogenetic changes in the pattern of androgen accumulation in
song-control nuclei of male zebra finches. J Neurobiol.

[b12] Bottjer SW (1993). The distribution of tyrosine hydroxylase immunoreactivity in the
brains of male and female zebra finches. J Neurobiol.

[b13] Bouthenet ML, Souil E, Martres MP, Sokoloff P, Giros B, Schwartz JC (1991). Localization of dopamine D3 receptor mRNA in the rat brain using
in situ hybridization histochemistry: comparison with dopamine D2 receptor
mRNA. Brain Res.

[b14] Bunzow JR, Van Tol HH, Grandy DK, Albert P, Salon J, Christie M, Machida CA, Neve KA, Civelli O (1988). Cloning and expression of a rat D2 dopamine receptor
cDNA. Nature.

[b15] Burge CB, Karlin S (1998). Finding the genes in genomic DNA. Curr Opin Struct Biol.

[b16] Callier S, Snapyan M, Le Crom S, Prou D, Vincent JD, Vernier P (2003). Evolution and cell biology of dopamine receptors in
vertebrates. Biol Cell.

[b17] Cardinaud B, Sugamori KS, Coudouel S, Vincent JD, Niznik HB, Vernier P (1997). Early emergence of three dopamine D1 receptor subtypes in
vertebrates. Molecular phylogenetic, pharmacological, and functional
criteria defining D1A, D1B, and D1C receptors in European eel Anguilla
anguilla. J Biol Chem.

[b18] Castelino CB, Ball GF (2005). A role for norepinephrine in the regulation of context-dependent
ZENK expression in male zebra finches (Taeniopygia guttata). Eur J Neurosci.

[b19] Castelino CB, Diekamp B, Ball GF (2007). Noradrenergic projections to the song control nucleus area X of
the medial striatum in male zebra finches (*Taeniopygia
guttata*. J Comp Neurol.

[b20] Casto JM, Ball GF (1994). Characterization and localization of D1 dopamine receptors in the
sexually dimorphic vocal control nucleus, area X, and the basal ganglia of
European starlings. J Neurobiol.

[b21] Choi WS, Machida CA, Ronnekleiv OK (1995). Distribution of dopamine D1, D2, and D5 receptor mRNAs in the
monkey brain: ribonuclease protection assay analysis. Brain Res Mol Brain Res.

[b22] Cornil CA, Castelino CB, Ball GF (2008). Dopamine binds to alpha(2)-adrenergic receptors in the song
control system of zebra finches (*Taeniopygia
guttata*. J Chem Neuroanat.

[b23] Dearry A, Gingrich JA, Falardeau P, Fremeau RT, Bates MD, Caron MG (1990). Molecular cloning and expression of the gene for a human D1
dopamine receptor. Nature.

[b24] Demchyshyn LL, Sugamori KS, Lee FJ, Hamadanizadeh SA, Niznik HB (1995). The dopamine D1D receptor. Cloning and characterization of three
pharmacologically distinct D1-like receptors from Gallus
domesticus. J Biol Chem.

[b25] Diaz J, Ridray S, Mignon V, Griffon N, Schwartz JC, Sokoloff P (1997). Selective expression of dopamine D3 receptor mRNA in
proliferative zones during embryonic development of the rat
brain. J Neurosci.

[b26] Dietl MM, Palacios JM (1988). Neurotransmitter receptors in the avian brain. I. Dopamine
receptors. Brain Res.

[b27] Ding L, Perkel DJ (2002). Dopamine modulates excitability of spiny neurons in the avian
basal ganglia. J Neurosci.

[b28] Ding L, Perkel DJ (2004). Long-term potentiation in an avian basal ganglia nucleus
essential for vocal learning. J Neurosci.

[b29] Doupe A, Perkel D, Reiner A, Stern E (2005). Birdbrains could teach basal ganglia research a new
song. Trends Neurosci.

[b30] Durstewitz D, Kroner S, Gunturkun O (1999). The dopaminergic innervation of the avian
telencephalon. Prog Neurobiol.

[b31] Dutar P, Vu HM, Perkel DJ (1998). Multiple cell types distinguished by physiological,
pharmacological, and anatomic properties in nucleus HVc of the adult zebra
finch. J Neurophysiol.

[b32] Feenders G, Liedvogel M, Rivas M, Zapka M, Horita H, Hara E, Wada K, Mouritsen H, Jarvis ED (2008). Molecular mapping of movement-associated areas in the avian
brain: a motor theory for vocal learning origin. PLoS One.

[b33] Fidler AE, van Oers K, Drent PJ, Kuhn S, Mueller JC, Kempenaers B (2007). Drd4 gene polymorphisms are associated with personality variation
in a passerine bird. Proc Biol Sci.

[b34] Gale SD, Perkel DJ (2005). Properties of dopamine release and uptake in the songbird basal
ganglia. J Neurophysiol.

[b35] Gale SD, Perkel DJ (2006). Physiological properties of zebra finch ventral tegmental area
and substantia nigra pars compacta neurons. J Neurophysiol.

[b36] Gale SD, Person AL, Perkel DJ (2008). A novel basal ganglia pathway forms a loop linking a vocal
learning circuit with its dopaminergic input. J Comp Neurol.

[b37] Gerfen CR (2000). Molecular effects of dopamine on striatal-projection
pathways. Trends Neurosci.

[b38] Gerfen CR, Engber TM, Mahan LC, Susel Z, Chase TN, Monsma FJ, Sibley DR (1990). D1 and D2 dopamine receptor-regulated gene expression of
striatonigral and striatopallidal neurons. Science.

[b39] Gerfen CR, Keefe KA, Gauda EB (1995). D1 and D2 dopamine receptor function in the striatum:
coactivation of D1-and D2-dopamine receptors on separate populations of
neurons results in potentiated immediate early gene response in
D1-containing neurons. J Neurosci.

[b40] Grandy DK, Zhang YA, Bouvier C, Zhou QY, Johnson RA, Allen L, Buck K, Bunzow JR, Salon J, Civelli O (1991). Multiple human D5 dopamine receptor genes: a functional receptor
and two pseudogenes. Proc Natl Acad Sci U S A.

[b41] Guivarc'h D, Vernier P, Vincent JD (1995). Sex steroid hormones change the differential distribution of the
isoforms of the D2 dopamine receptor messenger RNA in the rat
brain. Neuroscience.

[b42] Hackett S, Kimball R, Reddy S, Bowie R, Braun E, Braun M, Chojnowski J, Cox W, Han K, Harshman J (2008). A phylogenomic study of birds reveals their evolutionary history
science.

[b43] Hara E, Kubikova L, Hessler NA, Jarvis ED (2007). Role of the midbrain dopaminergic system in modulation of vocal
brain activation by social context. Eur J Neurosci.

[b44] Harding CF (2004). Brief alteration in dopaminergic function during development
causes deficits in adult reproductive behavior. J Neurobiol.

[b45] Harding CF, Barclay SR, Waterman SA (1998). Changes in catecholamine levels and turnover rates in
hypothalamic, vocal control, and auditory nuclei in male zebra finches
during development. J Neurobiol.

[b46] Heimovics SA, Riters LV (2008). Evidence that dopamine within motivation and song control brain
regions regulates birdsong context-dependently. Physiol Behav.

[b47] Heimovics SA, Cornil CA, Ball GF, Riters LV (2009). D1-like dopamine receptor density in nuclei involved in social
behavior correlates with song in a context-dependent fashion in male
European starlings. Neuroscience.

[b48] Hessler NA, Doupe AJ (1999). Social context modulates singing-related neural activity in the
songbird forebrain. Nat Neurosci.

[b49] Higgins DG, Sharp PM (1988). CLUSTAL: a package for performing multiple sequence alignment on
a microcomputer. Gene.

[b50] Hirano J, Archer SN, Djamgoz MB (1998). Dopamine receptor subtypes expressed in vertebrate (carp and eel)
retinae: cloning, sequencing and comparison of five D1-like and three
D2-like receptors. Receptors Channels.

[b51] Hurd YL, Suzuki M, Sedvall GC (2001). D1 and D2 dopamine receptor mRNA expression in whole hemisphere
sections of the human brain. J Chem Neuroanat.

[b52] Ilani T, Fishburn C, Levavi-Sivan B, Carmon S, Raveh L, Fuchs S (2002). Coupling of dopamine receptors to G proteins: studies with
chimeric D2/D3 dopamine receptors. Cell Mol Neurobiol.

[b53] Jarvis ED, Nottebohm F (1997). Motor-driven gene expression. Proc Natl Acad Sci U S A.

[b54] Jarvis ED, Scharff C, Grossman MR, Ramos JA, Nottebohm F (1998). For whom the bird sings: context-dependent gene
expression. Neuron.

[b55] Jarvis E, Güntürkün O, Bruce L, Csillag A, Karten H, Kuenzel W, Medina L, Paxinos G, Perkel D, Shimizu T, Striedter G, Wild JM, Ball GF, Dugas-Ford J, Durand SE, Hough GE, Husband S, Kubikova L, Lee DW, Mello CV, Powers A, Siang C, Smulders TV, Wada K, White SA, Yamamoto K, Yu J, Reiner A, Butler AB, Avian Brain Nomenclature Consortium (2005). Avian brains and a new understanding of vertebrate brain
evolution. Nat Rev Neurosci.

[b56] Kao MH, Brainard MS (2006). Lesions of an avian basal ganglia circuit prevent
context-dependent changes to song variability. J Neurophysiol.

[b57] Kao MH, Doupe AJ, Brainard MS (2005). Contributions of an avian basal ganglia-forebrain circuit to
real-time modulation of song. Nature.

[b58] Kapsimali M, Vidal B, Gonzalez A, Dufour S, Vernier P (2000). Distribution of the mRNA encoding the four dopamine D(1) receptor
subtypes in the brain of the european eel (*Anguilla
anguilla*): comparative approach to the function of D(1)
receptors in vertebrates. J Comp Neurol.

[b59] Kebabian JW, Calne DB (1979). Multiple receptors for dopamine. Nature.

[b60] Keefe KA, Gerfen CR (1995). D1–D2 dopamine receptor synergy in striatum: effects of
intrastriatal infusions of dopamine agonists and antagonists on immediate
early gene expression. Neuroscience.

[b61] Khan ZU, Gutierrez A, Martin R, Penafiel A, Rivera A, De La Calle A (1998). Differential regional and cellular distribution of dopamine
D2-like receptors: an immunocytochemical study of subtype–specific
antibodies in rat and human brain. J Comp Neurol.

[b62] Kitt CA, Brauth SE (1986). Telencephalic projections from midbrain and isthmal cell groups
in the pigeon. II. The nigral complex. J Comp Neurol.

[b63] Kleitz HK, Cornil CA, Balthazart J, Ball GF (2009). Species differences in the relative densities of D1-and D2-like
dopamine receptor subtypes in the Japanese quail and rats: an in vitro
quantitative receptor autoradiography study. Brain Behav Evol.

[b64] Kluger AN, Siegfried Z, Ebstein RP (2002). A meta-analysis of the association between DRD4 polymorphism and
novelty seeking. Mol Psychiatry.

[b65] Kostal L, Vyboh P, Savory CJ, Jurani M, Kubikova L, Blazicek P (1999). Influence of food restriction on dopamine receptor densities,
catecholamine concentrations and dopamine turnover in chicken
brain. Neuroscience.

[b66] Kubikova L, Turner EA, Jarvis ED (2007). The pallial basal ganglia pathway modulates the behaviorally
driven gene expression of the motor pathway. Eur J Neurosci.

[b67] Kubikova L, Vyboh P, Kostal L (2009). Kinetics and pharmacology of the D1-and D2-like dopaminer
receptors in Japanese quail brain. Cell Mol Neurobiol.

[b68] Kubota M, Taniguchi I (1998). Electrophysiological characteristics of classes of neuron in the
HVc of the zebra finch. J Neurophysiol.

[b69] Landwehrmeyer B, Mengod G, Palacios JM (1993a). Differential visualization of dopamine D2 and D3 receptor sites
in rat brain. A comparative study using in situ hybridization histochemistry
and ligand binding autoradiography. Eur J Neurosci.

[b70] Landwehrmeyer B, Mengod G, Palacios JM (1993b). Dopamine D3 receptor mRNA and binding sites in human
brain. Brain Res Mol Brain Res.

[b71] Le Crom S, Sugamori KS, Sidhu A, Niznik HB, Vernier P (2004). Delineation of the conserved functional properties of D1A, D1B
and D1C dopamine receptor subtypes in vertebrates. Biol Cell.

[b72] Le Moine C, Bloch B (1995). D1 and D2 dopamine receptor gene expression in the rat striatum:
sensitive cRNA probes demonstrate prominent segregation of D1 and D2 mRNAs
in distinct neuronal populations of the dorsal and ventral
striatum. J Comp Neurol.

[b73] Lee SP, So CH, Rashid AJ, Varghese G, Cheng R, Lanca AJ, O'Dowd BF, George SR (2004). Dopamine D1 and D2 receptor co-activation generates a novel
phospholipase C-mediated calcium signal. J Biol Chem.

[b74] Lewis MJ (1981). Age-related decline in brain stimulation reward: rejuvenation by
amphetamine. Exp Aging Res.

[b75] Macrae AD, Brenner S (1995). Analysis of the dopamine receptor family in the compact genome of
the puffer fish *Fugu rubripes*. Genomics.

[b76] Man PS, Wells T, Carter DA (2007). Egr-1-d2EGFP transgenic rats identify transient populations of
neurons and glial cells during postnatal brain development. Gene Expr Patterns.

[b77] Mansour A, Meador-Woodruff JH, Bunzow JR, Civelli O, Akil H, Watson SJ (1990). Localization of dopamine D2 receptor mRNA and D1 and D2 receptor
binding in the rat brain and pituitary: an in situ hybridization-receptor
autoradiographic analysis. J Neurosci.

[b78] Martens GJ, Molhuizen HO, Groneveld D, Roubos EW (1991). Cloning and sequence analysis of brain cDNA encoding a
*Xenopus* D2 dopamine receptor. FEBS Lett.

[b79] Meador-Woodruff JH, Mansour A, Healy DJ, Kuehn R, Zhou QY, Bunzow JR, Akil H, Civelli O, Watson SJ (1991). Comparison of the distributions of D1 and D2 dopamine receptor
mRNAs in rat brain. Neuropsychopharmacology.

[b80] Mello CV, Ribeiro S (1998). ZENK protein regulation by song in the brain of
songbirds. J Comp Neurol.

[b81] Mello CV, Jarvis ED, Denisenko N, Rivas M (1997). Isolation of song-regulated genes in the brain of
songbirds. Methods Mol Biol.

[b82] Mello CV, Vates GE, Okuhata S, Nottebohm F (1998). Descending auditory pathways in the adult male zebra finch
(*Taeniopygia guttata*. J Comp Neurol.

[b83] Momozawa Y, Takeuchi Y, Kusunose R, Kikusui T, Mori Y (2005). Association between equine temperament and polymorphisms in
dopamine D4 receptor gene. Mamm Genome.

[b84] Mooney R (2000). Different subthreshold mechanisms underlie song selectivity in
identified HVc neurons of the zebra finch. J Neurosci.

[b85] Mouritsen H, Feenders G, Liedvogel M, Wada K, Jarvis ED (2005). Night-vision brain area in migratory songbirds. Proc Natl Acad Sci U S A.

[b86] Nixdorf-Bergweiler B (2001). Lateral magnocellular nucleus of the anterior neostriatum (LMAN)
in the zebra finch: neuronal connectivity and the emergence of sex
differences in cell morphology. Microsc Res Techniq.

[b87] Noain D, Avale ME, Wedemeyer C, Calvo D, Peper M, Rubinstein M (2006). Identification of brain neurons expressing the dopamine D4
receptor gene using BAC transgenic mice. Eur J Neurosci.

[b88] Nordeen KW, Nordeen EJ (1993). Long-term maintenance of song in adult zebra finches is not
affected by lesions of a forebrain region involved in song
learning. Behav Neural Biol.

[b89] Nottebohm F, Arnold A (1976). Sexual dimorphism in vocal control areas of the songbird
brain. Science.

[b90] Nottebohm F, Nottebohm ME (1971). Vocalizations and breeding behaviour of surgically deafened ring
doves (*Streptopelia risoria*. Anim Behav.

[b91] Nottebohm F, Stokes TM, Leonard CM (1976). Central control of song in the canary, *Serinus
canarius*. J Comp Neurol.

[b92] Ölveczky B, Andalman A, Fee M (2005). Vocal experimentation in the juvenile songbird requires a basal
ganglia circuit. PLoS Biol.

[b93] Pace-Schott E, Hobson J (2002). The neurobiology of sleep: genetics, cellular physiology and
subcortical networks. Nat Rev Neurosci.

[b94] Panksepp J (2005). Affective consciousness: core emotional feelings in animals and
humans. Consciousness Cognit.

[b95] Person AL, Gale SD, Farries MA, Perkel DJ (2008). Organization of the songbird basal ganglia, including area
X. J Comp Neurol.

[b96] Rappaport MS, Sealfon SC, Prikhozhan A, Huntley GW, Morrison JH (1993). Heterogeneous distribution of D1, D2 and D5 receptor mRNAs in
monkey striatum. Brain Res.

[b97] Reiner A, Perkel DJ, Bruce LL, Butler AB, Csillag A, Kuenzel W, Medina L, Paxinos G, Shimizu T, Striedter G, Wild M, Ball GF, Durand S, Gunturkun O, Lee DW, Mello CV, Powers A, White SA, Hough G, Kubikova L, Smulders TV, Wada K, Dugas-Ford J, Husband S, Yamamoto K, Yu J, Siang C, Jarvis ED (2004a). Revised nomenclature for avian telencephalon and some related
brainstem nuclei. J Comp Neurol.

[b98] Reiner A, Perkel DJ, Mello CV, Jarvis ED (2004b). Songbirds and the revised avian brain
nomenclature. Ann N Y Acad Sci.

[b99] Reynolds JN, Wickens JR (2002). Dopamine-dependent plasticity of corticostriatal
synapses. Neural Netw.

[b100] Richfield EK, Young AB, Penney JB (1987). Comparative distribution of dopamine D-1 and D-2 receptors in the
basal ganglia of turtles, pigeons, rats, cats, and monkeys. J Comp Neurol.

[b101] Richfield EK, Penney JB, Young AB (1989). Anatomical and affinity state comparisons between dopamine D1 and
D2 receptors in the rat central nervous system. Neuroscience.

[b102] Rohrer B, Stell WK (1995). Localization of putative dopamine D2-like receptors in the chick
retina, using in situ hybridization and immunocytochemistry. Brain Res.

[b103] Rose SP, Stewart MG (1999). Cellular correlates of stages of memory formation in the chick
following passive avoidance training. Behav Brain Res.

[b104] Sakaguchi H, Saito N (1989). The acetylcholine and catecholamine contents in song control
nuclei of zebra finch during song ontogeny. Brain Res Dev Brain Res.

[b105] Salamone J (1992). Complex motor and sensorimotor functions of striatal and
accumbens dopamine: involvement in instrumental behavior
processes. Psychopharmacology.

[b106] Sasaki A, Sotnikova TD, Gainetdinov RR, Jarvis ED (2006). Social context-dependent singing-regulated
dopamine. J Neurosci.

[b107] Scharff C, Nottebohm F (1991). A comparative study of the behavioral deficits following lesions
of various parts of the zebra finch song system: implications for vocal
learning. J Neurosci.

[b108] Schnabel R, Braun K (1996). Development of dopamine receptors in the forebrain of the
domestic chick in relation to auditory imprinting. An autoradiographic
study. Brain Res.

[b109] Schnabel R, Metzger M, Jiang S, Hemmings HC, Greengard P, Braun K (1997). Localization of dopamine D1 receptors and dopaminoceptive neurons
in the chick forebrain. J Comp Neurol.

[b110] Schnell SA, You S, Foster DN, El Halawani ME (1999). Molecular cloning and tissue distribution of an avian D2 dopamine
receptor mRNA from the domestic turkey (*Maleagris
gallopavo*. J Comp Neurol.

[b111] Schroeder MB, Riters LV (2006). Pharmacological manipulations of dopamine and opioids have
differential effects on sexually motivated song in male European
starlings. Physiol Behav.

[b112] Schultz W (2007). Behavioral dopamine signals. Trends Neurosci.

[b113] So CH, Verma V, Alijaniaram M, Cheng R, Rashid AJ, O'Dowd BF, George SR (2009). Calcium signaling by dopamine D5–D2 receptor
heterooligomers occurs by a mechanism distinct from that for dopamine
D1–D2 receptor heterooligomers. Mol Pharmacol.

[b114] Soha JA, Shimizu T, Doupe AJ (1996). Development of the catecholaminergic innervation of the song
system of the male zebra finch. J Neurobiol.

[b115] Sohrabji F, Nordeen EJ, Nordeen KW (1990). Selective impairment of song learning following lesions of a
forebrain nucleus in the juvenile zebra finch. Behav Neural Biol.

[b116] Sokoloff P, Giros B, Martres MP, Bouthenet ML, Schwartz JC (1990). Molecular cloning and characterization of a novel dopamine
receptor (D3) as a target for neuroleptics. Nature.

[b117] Sossinka R, Prove E, Kalberlah HH (1975). [Effect of testosterone on the beginning of singing in the zebra
finch (*Taeniopygia guttata* castanotis)]. Z Tierpsychol.

[b118] Stewart MG, Kabai P, Harrison E, Steele RJ, Kossut M, Gierdalski M, Csillag A (1996). The involvement of dopamine in the striatum in passive avoidance
training in the chick. Neuroscience.

[b119] Stoof JC, Kebabian JW (1984). Two dopamine receptors: biochemistry, physiology and
pharmacology. Life Sci.

[b120] Sugamori KS, Demchyshyn LL, Chung M, Niznik HB (1994). D1A, D1B, and D1C dopamine receptors from *Xenopus
laevis*. Proc Natl Acad Sci U S A.

[b121] Sun Z, Reiner A (2000). Localization of dopamine D1A and D1B receptor mRNAs in the
forebrain and midbrain of the domestic chick. J Chem Neuroanat.

[b122] Sunahara RK, Niznik HB, Weiner DM, Stormann TM, Brann MR, Kennedy JL, Gelernter JE, Rozmahel R, Yang YL, Israel Y (1990). Human dopamine D1 receptor encoded by an intronless gene on
chromosome 5. Nature.

[b123] Sunahara RK, Guan HC, O'Dowd BF, Seeman P, Laurier LG, Ng G, George SR, Torchia J, Van Tol HH, Niznik HB (1991). Cloning of the gene for a human dopamine D5 receptor with higher
affinity for dopamine than D1. Nature.

[b124] Takeuchi Y, Fukunaga K (2004). Different activation of NF-κB by stimulation of dopamine
D2L and D2S receptors through calcineurin activation. J Neurochem.

[b125] Teramitsu I, White SA (2006). FoxP2 regulation during undirected singing in adult
songbirds. J Neurosci.

[b126] Vallone D, Picetti R, Borrelli E (2000). Structure and function of dopamine receptors. Neurosci Biobehav Rev.

[b127] Van Tol HH, Bunzow JR, Guan HC, Sunahara RK, Seeman P, Niznik HB, Civelli O (1991). Cloning of the gene for a human dopamine D4 receptor with high
affinity for the antipsychotic clozapine. Nature.

[b128] Wada K, Sakaguchi H, Jarvis ED, Hagiwara M (2004). Differential expression of glutamate receptors in avian neural
pathways for learned vocalization. J Comp Neurol.

[b129] Wada K, Howard JT, McConnell P, Whitney O, Lints T, Rivas MV, Horita H, Patterson MA, White SA, Scharff C, Haesler S, Zhao S, Sakaguchi H, Hagiwara M, Shiraki T, Hirozane-Kishikawa T, Skene P, Hayashizaki Y, Carninci P, Jarvis ED (2006). A molecular neuroethological approach for identifying and
characterizing a cascade of behaviorally regulated genes. Proc Natl Acad Sci U S A.

[b130] Weiner DM, Levey AI, Sunahara RK, Niznik HB, O'Dowd BF, Seeman P, Brann MR (1991). D1 and D2 dopamine receptor mRNA in rat brain. Proc Natl Acad Sci U S A.

[b131] Wirtshafter D (2007). Rotation and immediate-early gene expression in rats treated with
the atypical D1 dopamine agonist SKF 83822. Pharmacol Biochem Behav.

[b132] Wise RA (2004). Dopamine, learning and motivation. Nat Rev Neurosci.

[b133] Yanagihara S, Hessler NA (2006). Modulation of singing-related activity in the songbird ventral
tegmental area by social context. Eur J Neurosci.

[b134] Zawilska JB, Rosiak J, Berezinska M, Nowak JZ (2003). L-745,870 suppresses the nighttime serotonin N-acetyltransferase
activity in chick retina: in vivo evidence for agonist activity at
D4-dopamine receptors. J Neural Transm.

[b135] Zhou QY, Grandy DK, Thambi L, Kushner JA, Van Tol HH, Cone R, Pribnow D, Salon J, Bunzow JR, Civelli O (1990). Cloning and expression of human and rat D1 dopamine
receptors. Nature.

