# Supplementary figures and images for: Dopamine receptors in a songbird brain
Source: J Comp Neurol. 2010 Mar 15;518(6):741–69. doi: 10.1002/cne.22255 (PMC2904815; doi:10.1002/cne.22255)

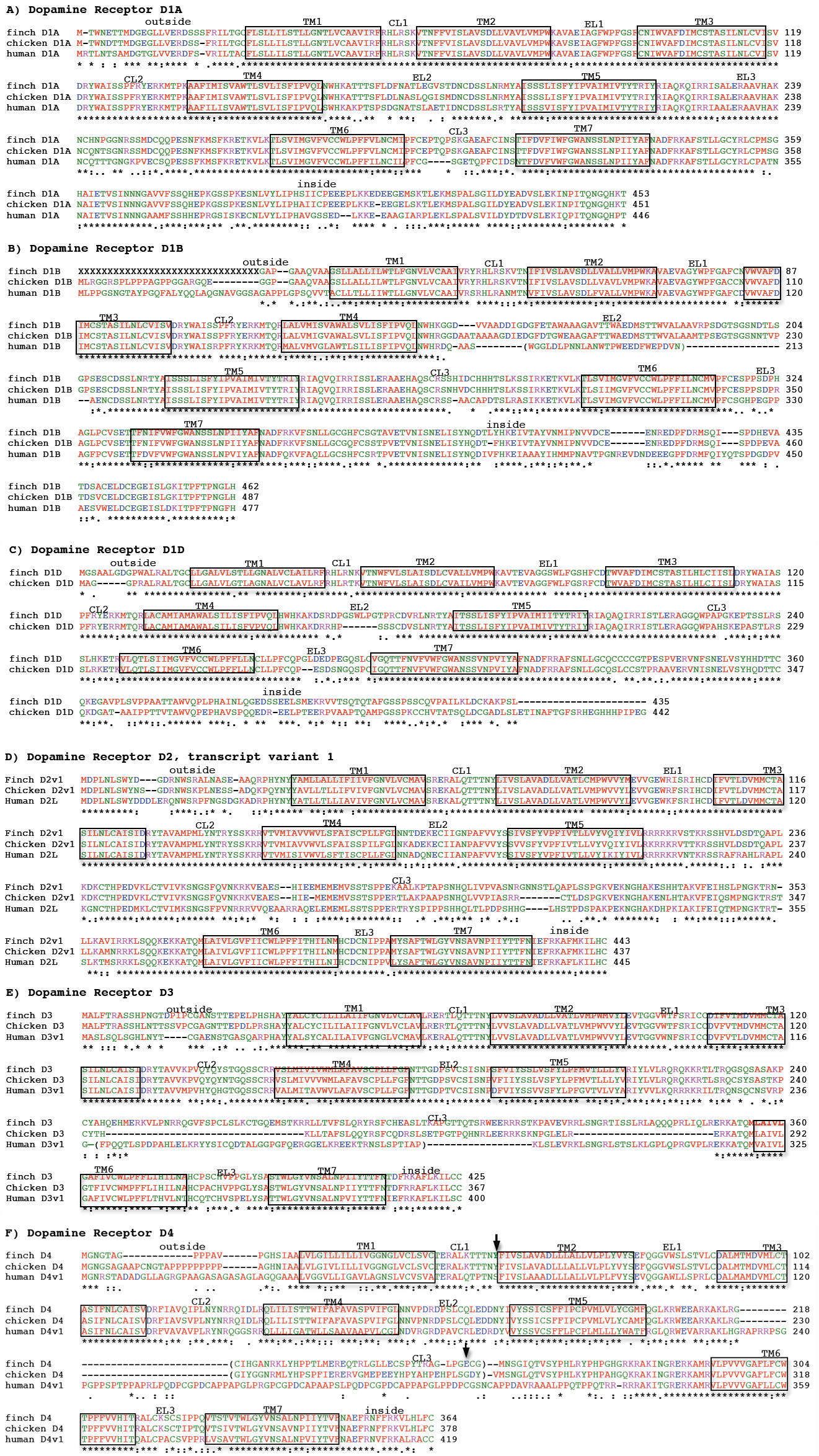

Supplement: Fig. S1 — Comparative protein sequence analyses of zebra finch dopamine receptor subtypes relative to chickens and humans. A-F: Receptor alignments for D1A, D1B, D1D, D2, D3, and D4, respectively. The protein coding sequences were aligned with clustalW www.ebi.ac.uk/Tools/clustalw2/ and then manually edited to correct errors in alignments. Text color coding: Red, small+hydrophobic, including aromatic (-Y) amino acids; Blue, acidic a.a.; Magenta, basic a.a.; Green, hydroxy+amine+basic (-Q) a.a.. Receptor domains were predicted with TMHMM software ( www.cbs.dtu.dk/services/TMHMM/) based on the zebra finch sequences. The exact predictions can slightly differ for different species, based on sequence differences. TM, transmembrane domain (boxed regions); CL, cytoplasmic loop; EL, extracellular loop. Outside and inside domains are the amino-and carboxy-terminal regions, respectively, that are positioned outside and inside of the cell, respectively. Note that the TM domains are mainly hydrophobic. For D1B (B), the first ∼23 a.a. of the zebra finch protein has not yet been sequenced from the zebra finch genome or cloned as a cDNA, and thus the sequence shown is partial. For D1D (C), the chicken sequence shown is the one annotated by some sources as D1C. For D2 (D), variant 1 for birds and the variant long of humans are aligned. For D3 (E), the prediction algorithms generated a longer protein at the amino terminal end in chicken than that supported by avian EST evidence and homologies to zebra finch and other vertebrate species (our analysis). Thus, we truncated the chicken sequence at the start site for zebra finch. The closest human D3 variant (variant 1) to the zebra finch protein was aligned. For D4, the zebra finch sequence between the arrows was determined from the cDNA clone of this study (part of which has not yet been sequenced yet in the genome), whereas the remaining sequence was determined from the genome. Accession numbers of the clones used are shown in Fig. 2. [file cne0518-0741-SD1.tif]

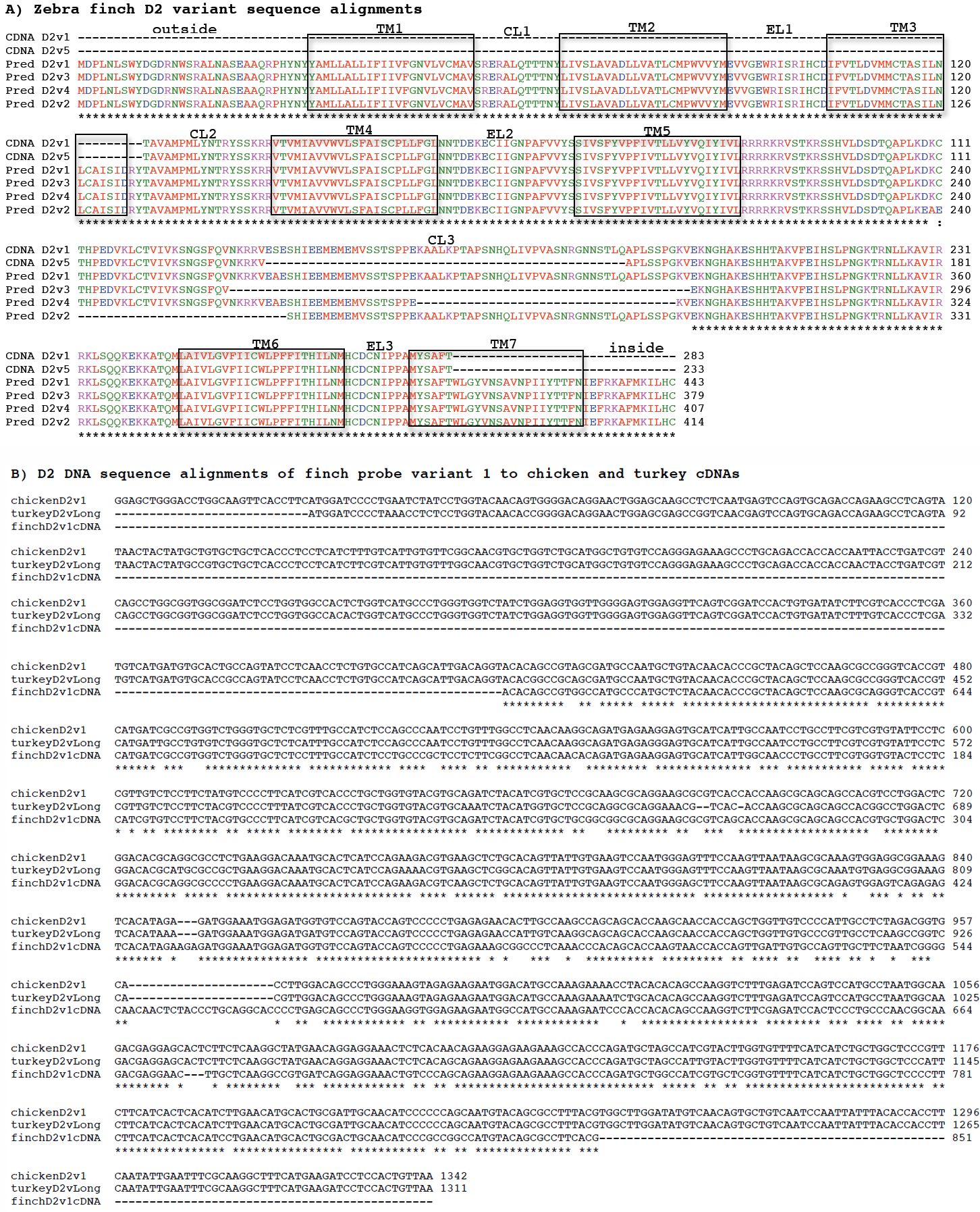

Supplement: Fig. S2 — Zebra finch D2 receptor variant alignments. A: Protein sequence alignments of cDNA supported and genomic predicted protein sequences of D2 splice variants. The cDNA inferred protein variants 1 and 5 (D2v1 and D2v5) were cloned in this study. The variants 3-4 (D2v1-D2v4) were predicted by ENSEMBLE and are in NCBI Genbank. Color-coding and labels follow the format described in the legend of Fig. S1. Note the splice variations in the 3rd cytoplasmic loop (CL3). B: Alignments of the zebra finch D2 variant 1 used for in situ hybridizations in this study with chicken D2 variant 1 and the turkey D2 long variant used by Schnell et al (1999). [file cne0518-0741-SD2.tif]

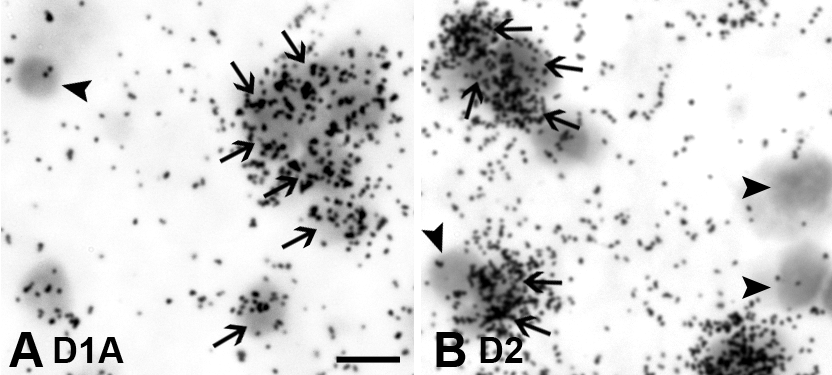

Supplement: Fig. S3 — Images from single label radioactive in-situ hybridization showing A: D1A and B: D2 receptor mRNA (silver grains in emulsion; black dots) above Nissl labeled cells (grey) in Area X of the striatum in zebra finch. Arrows, labeled cells; arrow heads, non-labeled cells. Scale bar, 10 μm. [file cne0518-0741-SD3.tif]
